# Deploying unsupervised clustering analysis to derive clinical phenotypes and risk factors associated with mortality risk in 2022 critically ill patients with COVID-19 in Spain

**DOI:** 10.1186/s13054-021-03487-8

**Published:** 2021-02-15

**Authors:** Alejandro Rodríguez, Manuel Ruiz-Botella, Ignacio Martín-Loeches, María Jimenez Herrera, Jordi Solé-Violan, Josep Gómez, María Bodí, Sandra Trefler, Elisabeth Papiol, Emili Díaz, Borja Suberviola, Montserrat Vallverdu, Eric Mayor-Vázquez, Antonio Albaya Moreno, Alfonso Canabal Berlanga, Miguel Sánchez, María del Valle Ortíz, Juan Carlos Ballesteros, Lorena Martín Iglesias, Judith Marín-Corral, Esther López Ramos, Virginia Hidalgo Valverde, Loreto Vidaur Vidaur Tello, Susana Sancho Chinesta, Francisco Javier Gonzáles de Molina, Sandra Herrero García, Carmen Carolina Sena Pérez, Juan Carlos Pozo Laderas, Raquel Rodríguez García, Angel Estella, Ricard Ferrer, on behalf of COVID-19 SEMICYUC Working Group, Ana Loza, Diego Matallana Zapata, Isabel Díaz Torres, Sonia Ibañez Cuadros, María Recuerda Nuñez, Maria Luz Carmona Pérez, Jorge Gómez Ramos, Alba Villares Casas, María Luisa Cantón, José Javier González Contreras, Helena Pérez Chomón, Nerissa Alvarez Chicote, Alberto Sousa González, María De Alba Aparicio, Juan Carlos Pozo Laderas, Angel Estella, Sara Moreno Cano, Diego Matallana Zapata, Ruth Jorge García, Laura Sánchez Montori, Sandra Herrero García, Paula Abanses Moreno, Carlos Mayordomo García, Tomás Mallor Bonet, Paula Omedas Bonafonte, Enric Franquesa Gonzalez, Nestor Bueno Vidales, Paula Ocabo Buil, Carlos Serón Arbeloa, Isabel Sancho, Pablo Guerrero Ibañez, Pablo Gutierrez, María Concepción Valdovinos, Raquel Canto, Ana Luz Balán Mariño, María José Gutiérrez Fernández, Marta Martín Cuadrado, Belén García Arias, Lorena Forcelledo Espina, Lucía Viña Soria, Lorena Martín Iglesias, Lucía López Amor, Elisabet Fernández Rey, Emilio García Prieto, Débora Fernández Ruíz, Carla Martínez González, Lorenzo Socias, Marcio Borges-Sá, María Aranda Pérez, Antonia Socias, José Ma Bonell Goytisolo, Inmaculada Alcalde Mayayo, Carlos Corradini, Isabel Ceniceros, Edwin Rodríguez, Jose Ignacio Ayestarán Rota, Mariana Andrea Novo Novo, Joaquim Colomina Climent, Albert Figueras Castilla, Tomàs Leal Rullan, Maria Magdalena Garcias Sastre, Rossana Pérez Senoff, Ramón Fernández-Cid Bouza, Juan Carlos Martín González, Carmen Pérez Ortiz, José Luciano Cabrera Santana, Juan José Cáceres Agra, Domingo González Romero, Ana Casamitjana Ortega, Luis Alberto Ramos Gómez, Jordi Solé-Violán, Alejandro Rodríguez, María Bodí, Gerard Moreno, Sandra Trefler, Laura Claverias, Raquel Carbonell, Erika Esteve, Montserrat Olona, Xavier Teixidó, Monserrat Vallverdú Vidal, Begoña Balsera Garrido, Elisabeth Papiol Gallofré, Raquel Albertos Martell, Rosa Alcaráz Peñarrocha, Xavier Nuvials Casals, Ricard Ferrer Roca, Eric Adrián Mayor Vázquez, Ferrán Roche Campo, Pablo Concha Martínez, Diego Franch Llasat, Joan Ramón Masclanz, Judith Marín-Corral, Purificación Pérez, Rosana Muñoz, Clara Vila, Francisco Javier González de Molina, Elisabeth Navas Moya, Josep Trenado, Imma Vallverdú, Eric Castañé, Emili Díaz Santos, Gemma Goma, Edgar Moglia, Borja Suberviola, Antonio Albaya Moreno, Carlos Marian Crespo, Carmen Carolina Sena Pérez, Francisca Arbol Linde, Diana Monge Donaire, Vega Losada Martínez, Nuria Rodrigo Castroviejo, Gerardo Ferrigno, Reyes Beltrán, Carolina Sanmartino, Concepción Tarancón Maján, Alfredo Marcos Gutiérrez, Virginia Hidalgo Valverde, Caridad Martín López, Oihane Badallo, María del Valle Ortiz, Rebeca Vara Arlanzón, David Iglesias Posadilla, María Teresa Recio, Juan Carlos Ballesteros, Enrique Laza Laza, Elena Gallego Curto, Ma Carmen Sánchez García, Miguel Díaz-Tavora, Rosa Mancha, Ana Ortega Montes, Isabel Gallego Barbachano, Eva Sanmartín Mantiñán, María Lourdes Cordero, Raquel María Rodríguez García, Jorge Gámez Zapata, María Gestal Vázquez, María José Castro Orjales, María Isabel Álvarez Diéguez, Carmen Rivero Velasco, Beatriz Lence Massa, María Gestal Vázquez, Ignacio Martínez Varela, Diego Matallana Zapata, Alberto Hernández Tejedor, Esther Ma López Ramos, Laura Alcázar Sánchez Elvira, Rocío Molina Montero, Ma Consuelo Pintado Delgado, María Trascasa MuñozMuñoz de la Peña, Yaiza Betania Ortiz de Zárate Ansotegui, Alejandra Acha Aranda, Juan Higuera Lucas, Juan Antonio Sanchez Giralt, Marta Chicot Llano, Nuria Arevalillo Fernández, Marta Sánchez Galindo, Ricardo Andino Ruiz, Alfonso Canabal Berlanga, Miguel Sánchez, Mercedes Nieto, Eduardo Arias Sarmiento, Adoración Bueno Blázquez, Rosa María de la Casa, Fátima Martín, Samuel González López, Elena Martínez Q uintana, Bernardo Gil Rueda, Áurea Higon Cañigral, Laura López Gómez, Pablo Safwat Bayoumi Delis, Augusto Montenegro Muore, Ángel Andrés Agamez Luengas, Enriqueta Andreu Soler, Ana Beatriz Pérez Pérez, José Higinio de Gea García, Rubén Jara Rubio, Silvia Sánchez Cámara, Alba Moreno Flores, José Moya Sánchez, Daniel Francisco Pérez Martínez, Ma Desamparados del Rey Carrión, María José Rico Lledó, Juana María Serrano Navarro, Juan Francisco Martín Ruíz, Julián Triviño Hidalgo, África López Ferrer, Isabel Cremades Navalón, Josefa Murcia Payá, JM Allegre Gallego, María del Carmen Lorente, Marta Gonsalvez, Ruth González Natera, Raquel Garrido López de Murillo, Tania Ojuel Gros, Raquel Flecha Viguera, Isabel López González, Adriana García Herrera, Loreto Vidaur Tello, Maialen Aseguinolaza, Itziar Eguibar, María Luisa Cantón Bulnes, Jose Javier González Contreras, Helena Pérez Chomón, Nerissa Álvarez Chicote, Alberto Sousa González, Asunción Marqués Parra, Sergio García Marti, Alberto Lorenzo Aguilar, Laura Bellver Bosch, Victor Gascón Sanchez, Sonia De la Guía Ortega, Martín Parejo Montell, Alberto Belenguer Muncharaz, Hector Hernández Garces, Victor Ramírez Montero, Mónica Crespo Gómez, Verónica Martí Algarra, Susana Sancho Chinesta, Joaquin Arguedas Cervera, Faustino Álvarez Cebrian, Begoña Balerdi Pérez, Rosa Jannone Fores, Javier Botella de Maglia, Nieves Carbonell Monleón, Jose Ferreres Franco, Ainhoa Serrano Lazaro, Mar Juan Díaz, María Luisa Blasco Cortés, Laura Fayos, Julia Giménez, Gaspar Soriano, Ricardo Navarro, Sonia Mas, Elena Bisbal, Laura Albert, Johncard Romero, Juan Fernández Cabreara, Andrea Ortíz, Antonio Margarit Ribas

**Affiliations:** 1 ICU Hospital Universitario Joan XXIII/IISPV/URV, Mallafre Guasch 4, 43007 Tarragona, Spain; 2 CIBERESUCICOVID, Barcelona, Spain; 3 Tarragona Health Data Research Working Group (THeDaR), ICU Hospital Universitario Joan XXIII, Tarragona, Spain; 4 Department of Intensive Care Medicine, Multidisciplinary Intensive Care Research Organization (MICRO), St. James’s Hospital, Dublin, Ireland; 5 Dean Nursing Faculty, Universitat Rovira i Virgili, Tarragona, Spain; 6 ICU Hospital Universitario Dr. Negrín, Las Palmas de Gran Canaria, Spain; 7 ICU Hospital Universitario Vall d’Hebron, Barcelona, Spain; 8 ICU Hospital Parc Tauli, Sabadell, Spain; 9 ICU Hospital Marqués de Valdecilla, Santander, Spain; 10 ICU Hospital Universitario Arnau de Vilanova, Lleida, Spain; 11 ICU Hospital Verge de la Cinta, Tortosa, Spain; 12 ICU Hospital Universitario de Guadalajara, Guadalajara, Spain; 13 ICU Hospital de La Princesa, Madrid, Spain; 14 ICU Hospital Clinico San Carlos, Madrid, Spain; 15 ICU Hospital Universitario de Burgos, Burgos, Spain; 16 ICU Hospital Clínico de Salamanca, Salamanca, Spain; 17 ICU Hospital Universitario Central de Asturias, Oviedo, Spain; 18 ICU Hospital del Mar, Barcelona, Spain; 19 ICU Hospital Príncipe de Asturias, Alcalá de Henares, Spain; 20 ICU Hospital Complejo Asistencial de Segovia, Segovia, Spain; 21 ICU Hospital Universitario de Donostia, Donosia, Spain; 22 ICU Hospital Universitario y Politécnico La Fe, Valencia, Spain; 23 ICU Hospital Universitario de Terrasa, Terrasa, Spain; 24 ICU Hospital Clínico Universitario Lozano Blesa, Zaragoza, Spain; 25 ICU Hospital Nuestra Señora del Prado, Talavera de la Reina, Spain; 26 ICU Hospital Universitario Reina Sofía, Córdoba, Spain; 27 ICU Complejo Hospitalario Universitario a Coruña, A Coruña, Spain; 28 ICU Hospital Universitario de Jerez, Jerez de la Frontera, Spain

**Keywords:** Severe SARS-CoV-2 infection, Phenotypes, Risk factors, Prognosis, Machine learning

## Abstract

**Background:**

The identification of factors associated with Intensive Care Unit (ICU) mortality and derived clinical phenotypes in COVID-19 patients could help for a more tailored approach to clinical decision-making that improves prognostic outcomes.

**Methods:**

Prospective, multicenter, observational study of critically ill patients with confirmed COVID-19 disease and acute respiratory failure admitted from 63 ICUs in Spain. The objective was to utilize an unsupervised clustering analysis to derive clinical COVID-19 phenotypes and to analyze patient’s factors associated with mortality risk. Patient features including demographics and clinical data at ICU admission were analyzed. Generalized linear models were used to determine ICU morality risk factors. The prognostic models were validated and their performance was measured using accuracy test, sensitivity, specificity and ROC curves.

**Results:**

The database included a total of 2022 patients (mean age 64 [IQR 5–71] years, 1423 (70.4%) male, median APACHE II score (13 [IQR 10–17]) and SOFA score (5 [IQR 3–7]) points. The ICU mortality rate was 32.6%. Of the 3 derived phenotypes, the A (mild) phenotype (537; 26.7%) included older age (< 65 years), fewer abnormal laboratory values and less development of complications, B (moderate) phenotype (623, 30.8%) had similar characteristics of A phenotype but were more likely to present shock. The C (severe) phenotype was the most common (857; 42.5%) and was characterized by the interplay of older age (> 65 years), high severity of illness and a higher likelihood of development shock. Crude ICU mortality was 20.3%, 25% and 45.4% for A, B and C phenotype respectively. The ICU mortality risk factors and model performance differed between whole population and phenotype classifications.

**Conclusion:**

The presented machine learning model identified three clinical phenotypes that significantly correlated with host-response patterns and ICU mortality. Different risk factors across the whole population and clinical phenotypes were observed which may limit the application of a “one-size-fits-all” model in practice**.**

## Introduction

Since the outbreak of COVID-19 disease began in December 2019 in China, soaring cases of confirmed SARS-CoV-2 are pummeling the global health system. More than 91 million people have developed SARS-CoV-2 infection, and more than 2 million have died [1]. Critical illness from COVID-19 has constrained intensive care unit (ICU) material and human resources [2]. As of January 18, 2021 more than 2.5 million people in Spain have been infected with SARS-CoV-2 and more than 53,000 have died [3]. Short-term mortality reported rate ranges from 16 to 62% of patients admitted to ICU [4–8]. The heterogeneity of patients that have been treated in China [4], Italy [8], USA [5–7] or Spain [9, 10] may explain the wide variation of mortality rate due to their population characteristics, presence of comorbidities and healthcare systems. A recent international survey [11] reported significant practice variations in the management of severe COVID-19 patients, including differences at the regional, hospital, and patient level. Therefore, it is necessary to characterize phenotypes, by extending the enrolment of patients outside of one ICU site to multiple patients being treated in different hospitals. Allowing to adequately measure mortality-related factors adjusted by the inter-hospital variation to determine clinical outcomes.

Risk factors represent the most important approach when defining treatment of hospitalized patients as these measures can inform clinical courses most likely for a patient given their a priori risk. However, risk factors can also interplay differently when they are included in different patient clusters. A single model based on general risk factors (one-size-fits-all) might be limited for clinical data interpretation and application across sites. Different combinations of risk factors may naturally cluster into previously undescribed subsets or phenotypes that may have different risks for a high mortality rate and that may therefore help to determine the response to treatments in COVID-19. We hypothesize that the presence of well-defined phenotypes in COVID-19 could help to more appropriately identify patients at risk of ICU mortality than general models for the entire population considering that this disease results in a constellation of symptoms, laboratory derangement, immune dysregulation, and clinical complications.

The primary objective was to determine the presence of distinct clinical phenotypes using unsupervised clustering methods that were applied to the datasets available on ICU admission. The second objective was to assess which factors are independently associated with ICU mortality. The added value of this large-scale multicenter prospective study lies to discover phenotypes based on clinical data available at ICU admission that can help explain the variation in clinical results of COVID-19 disease in the ICU.

## Material and methods

### Study design

A multicenter observational, prospective cohort study that consisted of a large-scale data source of hospital ICU admissions and patient-level clinical data. The enrolment criteria included adult’ patients with laboratory confirmed SARS-CoV-2 infection admitted in 63 ICUs across Spain due to acute respiratory failure between February 22, 2020 and May 11, 2020. The study was approved by the reference institutional review board at Joan XXIII University Hospital (IRB# CEIM/066/2020) and each participating site with a waiver of informed consent. All data values were anonymized prior to the phenotyping which consisted of clustering clinical variables on their association with COVID-19 mortality.

### Study sites and patients population

The study enrolled consecutive adult patients (> 16 years) with laboratory confirmed SARS-CoV-2 infection, detected by RT-PCR positive test of nasopharyngeal, oropharyngeal swab or invasive respiratory samples according to the WHO recommendations [12]. The follow-up of patients was scheduled until August 11, 2020, which confirmed ICU discharge or death whichever occurred first. A complete list of participating ICUs and their investigators is provided in the acknowledgements section. In this cohort, 43 patients were described in a preliminary report of a single–center case series in Tarragona, Spain [9].

### Outcomes

The primary outcome included all-causes of ICU mortality. Patients who were discharged alive from ICU were evaluated in the data as alive considering mortality was defined as any in-ICU death. All complications and outcomes were followed during ICU admission.

### Data collection

Data was obtained from a voluntary registry created by Spanish Society of Intensive Care Medicine-SEMICYUC. All consecutive cases admitted to the ICU were collected. There were no patients excluded from the analysis that was enrolled to participating ICU and met criteria.

All the collected variables recorded at ICU admission are listed in the Additional file 1: p. 6. To determine severity of illness, the Acute Physiology and Chronic Health Evaluation (APACHE) II score [13] and Sequential Organ Failure Assessment (SOFA) scoring [14] were calculated for all patients within the first 24 h of ICU admission.

The ICU admission criteria, use of antiviral, antibiotic or co-adjuvant treatment, and also the measures that would determine the need to intubate and type of ventilator support required (oxygenation, high flow nasal cannula [HFNC], noninvasive [NIV] or invasive [IMV] mechanical ventilation) were not standardized between centers and were left to the discretion of the attending physician, according to SEMICYUC and National Ministry of Health [15] and were included in the case report form and confirmed by the medical records. We also collected hospital-level data including city, county and number of hospital beds available. The study definitions used in the present analysis are shown in the Additional file 1: p. 2.

### Statistical analysis

No statistical sample size calculation was performed a priori, and sample size was equal to the number of patients admitted to the participant’s ICUs with confirmed COVID-19 during the study period. To describe baseline characteristics, the continuous variables were expressed as median (interquartile range [IQR]) and categorical variables as number of cases (percentage). For patient demographics and clinical characteristics, differences between groups were assessed using the chi-squared test and Fisher’s exact test for categorical variables, and the Mann–Whitney U or Wilcoxon test for continuous variables. To performed the analysis, we first assessed the candidate variables, missing values, and correlation. Multiple imputation was used to account for missing data (Additional file 1: p. 2). After evaluating correlation, highly correlated variables were excluded (Additional file 1: p. 5).

**Fig. 1:**
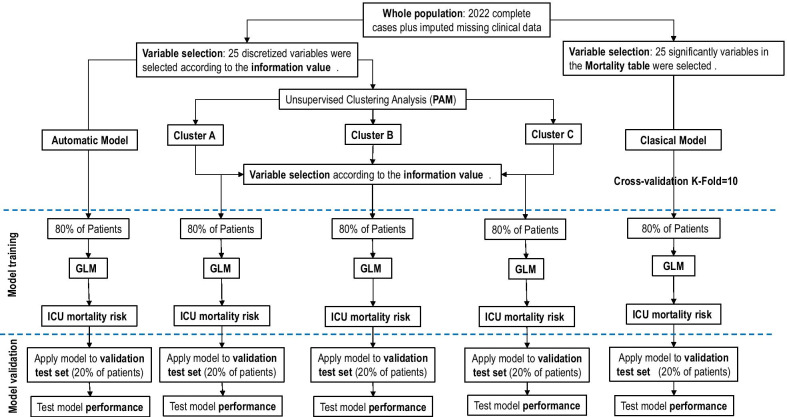
Overview of the primary analysis plan. *ICU* Intensive care units, *PAM* partition around medoids clustering analysis, *GLM* Generalized Linear model

An overview of the primary analysis plan is outlined in Fig. 1. In a first step, a multilevel conditional logistic modelling and the intraclass correlation coefficient (ICC) was calculated (Additional file 1: p. 2) with patients nested in hospital to characterize hospital-level variation of ICU mortality and determine if a significant inter-hospital variation is present.

In a second step, to determine presence of distinct clinical phenotypes in our population of COVID-19 patients, an unsupervised clustering analysis was applied to the database at ICU admission. In order to carry out this analysis, a discretization of the numerical variables into categorical ones was done using “ChiMerger” packages for R software. The information provided by each variable regarding ICU mortality was defined using the Information Value (IV). A IV greater than 0.03 was considered clinically important and this variable was included in the multivariate logistic regression analysis. Model performance was examined using accuracy test, Sensitivity, Specificity and AUC modeling. Subsequently, the unsupervised cluster analysis was performed using the important variables. The Podani distance was used to calculate the distance between patients and the “partition around medoids” (PAM) algorithm to perform the clustering [16]. The optimal number of clusters were determined after studying the silhouette [17] and the PAM objective for different numbers of clusters (Additional file 1: p. 12). Each of these clusters represent a specific patient’s phenotype. To visualize the clusters in a lower dimensional space, we used a Principal Component Analysis (PCA). We obtain important variables according to IV for each phenotype, and the OR of these variables were obtained after applying a GLM (Generalize linear Regression model) analysis. GLM is how statistical software R performs multiple logistic regression analysis when the command family = “binomial” is indicated. Multinomial regression models were fit to further compare patient comorbidities across phenotype classification. Model performance in each phenotype was examined using accuracy test, Sensibility, Specificity and AUC.

Lastly, a traditional multivariate analysis GLM was performed to investigate the association between baseline (on ICU admission) variables and ICU-mortality. The GLM model comprised factors of clinical interest and all significant covariates (*p* < 0.05) in the univariate analysis of ICU mortality and presence of collinearity was studied by variance inflation factors (VIF). The results are presented as odds ratios (OR) and 95% confidence intervals (CI). To determine our model, we checked adequate model performance between groups with a cross validation model (K-fold = 10) and the model with better performance was chosen.

For all model validation, database was randomly split into two subsets: (a) a “training set” (80%), and (b) a “validation set” (20%). Model performance was examined using accuracy test, precision, sensitivity, specificity and area under ROC curve (AUC). Data analysis was performed using R software (cran.r-project.org).

## Results

### Patients characteristics at ICU admission

From February 29, 2020 to June 11, 2020 a total of 2,022 critically ill patients from 63 ICUs were enrolled in the present analysis. Forty percent of ICUs belonged to hospitals with more than 500 beds, 40% to hospitals between 200 and 500 beds and the remaining 20 percent to hospitals with fewer than 200 beds. To determine if a significant inter-hospital variation is present, multilevel conditional logistic modelling with patients nested in hospital to characterize hospital-level variation of ICU mortality was done. According to intraclass correlation coefficient (ICC) obtained 0.04 when considering all hospital (n = 63) and of 0.04 when excluded hospitals that submitted data on few than 10 patients, no significant inter-hospital variation was observed (Additional file 1: e-Fig. 2, p. 7).

**Fig. 2:**
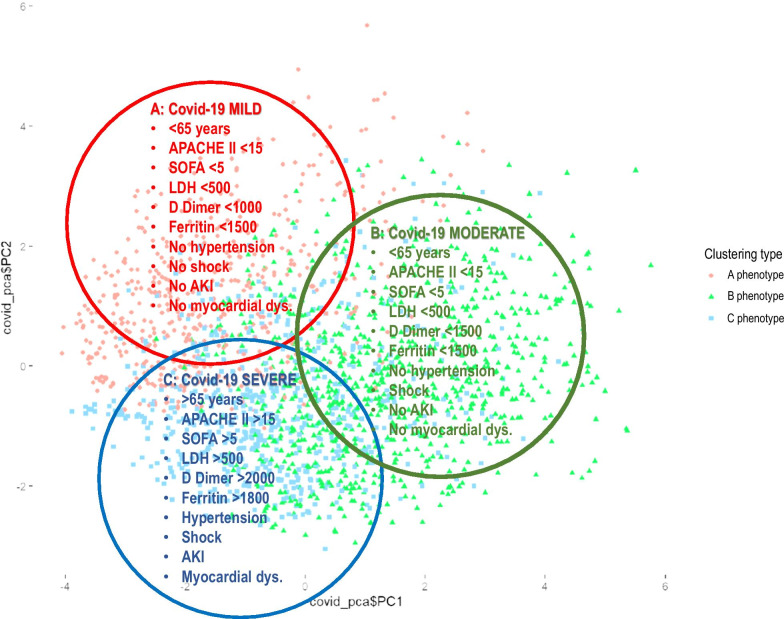
Phenotype clinical characterization (*APACHE II* Acute Physiology and Chronic Health Evaluation II, *SOFA* Sequential Organ Failure Assessment, *LDH* D-Lactate dehydrogenase, U/L, *AKI* Acute Kidney injury)

The median (IRQ) age was 64 (55–71) years, and 1,423 (70.3%) were men. The median of time between the onset of symptoms and diagnosis was 7 (4–9) days. A total of 1,467 (72.6%) patients had at least one coexisting comorbidity. Arterial hypertension (936 [46.7%]), obesity (655 [32.4%]) and diabetes mellitus (420 [20.7%]) were the most frequently comorbid conditions reported. The severity of illness was high according to the APACHE II (14; IQR 11–18) and SOFA (5.7; IQR 4–7.3) scores. The PaO_2_/FiO_2_ ratio on the day of ICU admission was 132(IQR 96–163) and 1131 (55·9%) patients meet criteria of severe and 198(9·8%) of mild acute distress respiratory syndrome (ADRS) profile at ICU admission, 1174 (58.0%) patients required invasive mechanical ventilation (MV), 906 (44.8%) developed shock and 580 (28.7%) meet criteria of acute kidney injury (AKI). The overall ICU length of stay (LOS) was 14 (8–26) days and similar for survivors (14 [8–27]) days and non-survivors (14 [8–24]) days (*p* = 0.10).

The most frequent prescribed co-adjuvant treatments for COVID-19-related infection were hydroxychloroquine (1866 [92.3%]) and lopinavir/ritonavir (1662 [82.2%]). Empiric antibiotic treatment was administered in 1818 (89.9%) of the patients and intravenous corticosteroids in 1174 (58.6%). Further clinical characteristics of patients and laboratory finding are shown in Additional file 1: e-Table 3, p. 8.

### ICU mortality

Overall, 660 patients (32.6%) died. The crude ICU mortality increased significantly with the increase in predefined age cut-off and was greater than 80% in patients over 80 years old (Additional file 1: p. 7). Age, male sex, severity of illness (APACHE II and SOFA), presence of arterial hypertension, diabetes, coronary arterial disease, chronic obstructive pulmonary disease (COPD), chronic kidney disease (CKD), immunosuppression and hematologic disease markers were significantly higher in ‘non-survivor’ patients. Non- survivor patients compared with those that survived had higher levels of D-Lactate dehydrogenase (LDH), white blood cells, serum creatinine, C-reactive protein (CRP), Procalcitonin (PCT), serum lactate, serum D-dimer and serum ferritin. Non-survivor patients developed more frequent complications such as shock, kidney and myocardial dysfunction at ICU admission. High Flow Nasal Cannula (HFNC) was more frequent in survivors, while invasive mechanical ventilation (MV) was more common in non-survivors. Mortality for those who received MV during their ICU stay (n = 1554; 76.8%) was 37.3% (n = 580) higher than observed in patients who did not require MV 17.0% (80/468, *p* < 0.001). Complete characteristics of patients according outcome are shown in Table 1.

**Table 1:** Characteristics of 2022 patients according ICU outcome

Variable^a,l^	Survival	No survival	*p* value
*General characteristics and severity of illness*			
No. of patients (%)	1362 (67.4)	660 (32.6)	
Age, median (IQR), y	61 (53–69)	69 (63–74)	.001
Male, No. (%)	939 (68.9)	484 (79.8)	.001
APACHE II^b^, median (IQR)	13 (10–17)	17 (12–21)	.001
SOFA^c^, median (IQR)	5 (3–7)	7 (5–8)	.001
*Laboratory findings*			
D-Lactate dehydrogenase, median (IQR), U/L	507 (397–667)	600 (475–808)	.001
White blood cell, median (IQR), × 10^9^	8.4 (6.1)	9.7 (6.6–13.6)	.001
Serum Creatinine, median (IQR), mg/dL	0.8 (0.6–1.0)	0.9 (0.7–1.3)	.001
C-Reactive Protein, median (IQR) mg/mL	15 (8.7–23.5)	17 (10.2–26.4)	.001
Procalcitonin, median (IQR),ng/mL	0.3 (0.1–0.7)	0.5 (0.2–1.4)	.001
Serum lactate, median (IQR) mmol/L	1.4 (1.1–1.9)	1.7 (1.3–2.4)	.001
D dimer, median (IQR), ng/mL	1280 (634–2900)	2230 (1000–5190)	.001
Ferritin median (IQR), ng/mL	1530 (1200–2000)	1900 (145–2500)	.001
*Treatments*			
Corticosteroids, No. (%)	777 (57.0)	397 (65.5)	.20
Antibiotics, No. (%)	1223 (89.8)	595 (98.1)	.86
Lopinavir/ritonavir, No. (%)	1111 (81.6)	551 (90.9)	.32
Hydroxychloroquine, No. (%)	1268 (93.1)	598 (98.6)	.06
Tocilizumab, No. (%)	407 (29.9)	170 (28.0)	.06
Interferon β, No. (%)	456 (33.5)	259 (42.7)	.01
*Coexisting condition and Comorbidities*			
Arterial hypertension, No. (%)	561 (41.2)	375 (61.9)	.001
Obesity^d^, No. (%)	431 (31.6)	224 (37.0)	.32
Diabetes, No. (%)	245 (17.9)	175 (28.8)	.001
Coronary arterial disease, No. (%)	58 (4.2)	66 (10.9)	.001
COPD, No. (%)	74 (5.4)	74 (12.2)	.001
Chronic renal disease^e^, No. (%)	42 (3.0)	43 (7.0)	.001
Hematologic disease^f^, No. (%)	36 (2.6)	37 (6.1)	.001
Asthma, No. (%)	83 (6.0)	38 (6.3)	.84
HIV, No. (%)	4 (0.3)	1 (0.1)	.89
Pregnancy, No. (%)	4 (0.3)	0 (0.0)	.38
Autoimmune disease, No. (%)	48 (3.5)	26 (4.3)	.73
Chronic heart disease^g^, No. (%)	34 (2.5)	23 (3.8)	.26
Neuromuscular disease, No. (%)	7 (0.5)	9 (1.5)	.07
Other immunosuppression^h^, No. (%)	25 (1.8)	28 (4.6)	.002
*Oxygenation and ventilator support*			
PaO_2_/FiO_2_, median(IQR), mmHg	135 (101–170)	121 (85–151)	.001
PaO_2_/FiO_2_ < 150 mmHg, n (%)	814 (59.7)	475 (71.9)	.01
Oxygen mask, No. (%)	235 (17.2)	93 (15.3)	.08
High Flow nasal cannula, No. (%)	295 (21.6)	80 (13.2)	.001
Non-invasive ventilation, No. (%)	98 (7.2)	42 (6.9)	.55
Invasive mechanical ventilation, No. (%)	716 (52.2)	458 (75.5)	.001
*Complications*			
Shock^i^, No. (%)	539 (39.6)	367 (60.5)	.001
Acute kidney dysfunction^j^, No. (%)	258 (18.9)	322 (53.1)	.001
Community-acquired co-infection^k^, No. (%)	117 (8.6)	73 (12.0)	.08
> 2 Quadrant infiltrates in chest x-ray, No. (%)	865 (63.5)	468 (77.2)	.001
Cardiac dysfunction, No. (%)	73 (5.3)	96 (15.8)	.001

*IQR* interquartile range, *APACHE II* Acute Physiology and Chronic Health Evaluation II, *SOFA* Sequential Organ Failure Assessment, *BMI* body mass index, *COPD* Chronic obstructive pulmonary disease, *HIV* human immunodeficiency viruses, *PaO*_*2*_*/FiO*_*2*_ Partial pressure arterial oxygen/fraction of inspired oxygen

^a^Corresponds to minimum or maximum value, as appropriate, within 12 h of ICU admission. The variables in this Table were no transformed for your comparison

^b^APACHE II score to the severity of illness, the score is obtained by adding the following components (1) 12 clinical and laboratory variables each with a score range of 0 to 4 points (APS). The APS is determined from the worst physiologic values during the initial 24 h after ICU admission, (2) age with a range 0 to 6 points and (3) Chronic health points if the patients has history of severe organ system insufficiency or is immunocompromised assign 5 points if the patients is no operative or emergency postoperative and 2 points for elective postoperative patients with a total score range of 0 to 71

^c^SOFA score corresponds to the severity of organ dysfunction, reflecting six organ systems each with a score range of 0 to 4 points (cardiovascular, hepatic, hematologic, respiratory, neurological, renal), with a total score range of 0 to 24

^d^Defined as a body mass index (calculated as weight in kilograms divided by height in meters squared) of 30 or greater

^e^Baseline eGFR < 60 on at least two consecutive values at least 12 weeks apart prior or hemodialysis

^f^Included acute leukemia, myelodysplastic syndrome and Lymphomas

^g^According to the New York Heart Association (NYHA) Functional Classification III and IV

^h^Included Chronic corticosteroid treatment (> 20 mg prednisolone/day or equivalent dose), chemotherapy or therapy with biological agents

^I^Defined as patients in whom adequate fluid resuscitation therapy are unable to restore hemodynamic stability and need any dose of vasopressor drugs

^j^Define as an abrupt and sustained (more than 24 h) decrease in kidney function and categorized according to RIFLE criteria

^k^Was considered in patients with confirmation of SARS-CoV-2 infection showing recurrence of fever, increase in cough and production of purulent sputum plus positive bacterial/fungal respiratory or blood cultures at ICU admission

^l^Kruskal-Wallis, ANOVA, or chi-square *p* value as appropriate comparing survivors vs. non-survivors

### Unsupervised analysis (cluster) to determine different phenotypes in critically ill patients

**Fig. 3:**
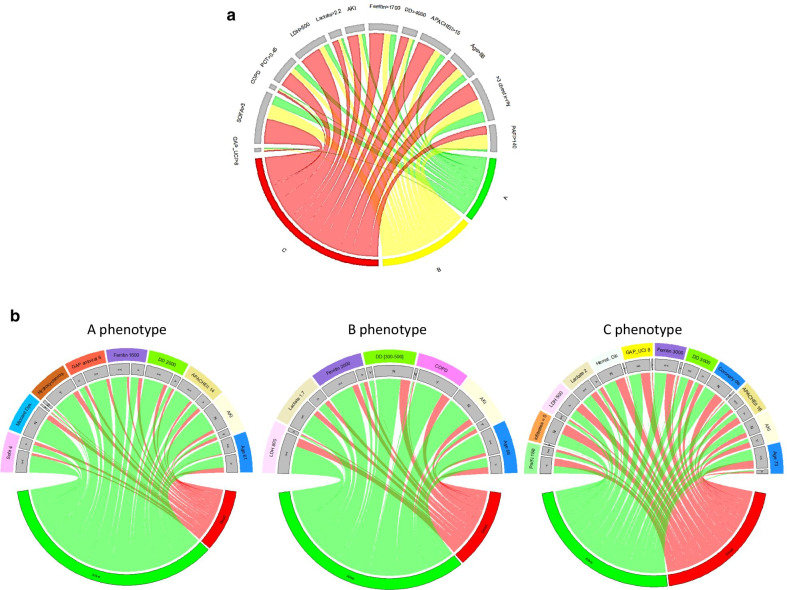
**a** Chord diagrams showing abnormal clinical variables by phenotype. A: mild COVID-19 disease; B: moderate COVID-19 disease and C: severe COVID-19 disease. **b** Chord diagrams showing abnormal clinical variables by Phenotype differentiating survivors (green) from non-survivors (red) (*APACHE II* Acute Physiology and Chronic Health Evaluation II, *SOFA* Sequential Organ Failure Assessment, *PCT* Procalcitonin, > *3 chest X-ray* more than 3 quadrants infiltrates in the chest X-ray, *Miocard Dys* Myocardial dysfunction, *Hydroxichloroq.* Hydroxychloroquine, *GAP antiviral* Time in days from onset of symptoms to first dose of antiviral, *DD* D dimer, *AKI* Acute Kidney injury, *LDH* D-Lactate dehydrogenase, U/L, *COPD* Chronic Pulmonary Obstructive Disease, *Pa/Fi* Partial pressure arterial oxygen/fraction of inspired oxygen, *Hemat. Dis* Hematologic disease, *GAP_UCI* Time in days from Hospital to ICU admission, *Coronary dis.* Coronary disease)

Once the variables were categorized, 5 patients (0.24%) were excluded for outlier’s data, and the analysis was performed with 2,017 patients. Of the 50 variables considered, only 25 were considered as predictors according to the IV (Additional file 1: p. 11) and were included in the model. Remarkably, no treatment option was a predictive factor for ICU-mortality. The categorized variables independently associated with ICU-mortality are shown in Additional file 1: p. 12. The performance of the model was adequate with an accuracy of 0.77, sensitivity of 0.88, specificity of 0.54 and AUC of 0.82. According to the Podani’s distance and the Shilouette and PAM plots (Additional file 1: p. 13) the optimal number of clusters in our dataset was 3. Cluster A included 537 patients (26.7%), cluster B included 623 (30.8%) and cluster C included 857 patients (42.5%). The clusters in a lower dimensional space are shown in the Additional file 1: p. 14. The size and characteristics of the phenotypes in the 3-class model are shown in Table 2. Patients with the cluster A phenotype (mild COVID-19 disease) had < 65 years, lower severity of illness, fewer abnormal laboratory values and less development of complications, with a crude ICU mortality of 20.3%; those with the cluster B phenotype (moderate COVID-19 disease) had similar characteristics as seen in the A phenotype but were more likely to present shock at ICU admission with a crude ICU-mortality of 25.5%. Patients with the cluster C phenotype (severe COVID-19 disease) had > 65 years, a high level of severity of illness, more likely to have elevated measures of inflammation (e.g. D dimer, LDH and ferritin), high frequency of shock, AKI and myocardial dysfunction, with a crude ICU mortality of 45.4%. The clinical characterization of each observed phenotype can be seen in Fig. 2. By including these important variables in a regression model for each cluster, we observed that the discrimination of each model was higher than general model except for C phenotype (Table 3). The Variables independently associated with mortality were different between automatic and cluster models (Table 4 and Fig. 3 A-B).

**Table 2:** Characteristics of 2017 critically ill patients included in machine learning analysis according to overall or cluster (phenotype) population

Variable^a^	Overall *n* = 2017	Cluster C (severe) *n* = 857	Cluster B (moderate) *n* = 623	Cluster A (mild) *n* = 537
*General characteristics and severity of illness*				
Age, mean (IQR), years	64 (55–71)	66 (58–72)	63 (53.5–71.5)	63 (53–70)***
Male, *n* (%)	1419 (70.3)	626 (73.0)	416 (66.8)	377 (70.2)*
APACHE II, mean (IQR)^b^	13 (10–17)	17 (14–22)	13 (10–16)	12 (9–16)***
SOFA, mean (IQR)^c^	5 (3.7)	7 (6–8)	5 (3–7)	4 (3–5)***
*Laboratory findings*				
D-Lactate dehydrogenase, mean (IQR), U/L	537 (417–707)	670 (554–929)	477 (378–570)	474 (372–564)***
White blood cell, mean (IQR), × 10^9^	8.8 (6.2–12.2)	10 (6.9–13.6)	8.5 (6–11.7)	7.7 (5.8–10.2)***
Serum Creatinine, mean (IQR), mg/dL	0.88 (0.7–1.1)	0.99 (0.76–1.36)	0.80 (0.66–1.00)	0.80 (0.66–1.01)***
C-Reactive Protein, mean (IQR), mg/mL	15.5 (9.1–24.3)	18 (10–26)	14 (9–22)	14 (8–2)***
Procalcitonin, mean (IQR), ng/mL	0.3 (0.1–2.0)	0.5 (0.2–1.3)	0.2 (0.1–0.5)	0.2 (0.1–0.6)***
Serum lactate, mean (IQR), mmol/L	1.5 (1.1–2.0)	1.6 (1.2–2.2)	1.4 (1.0–1.9)	1.5 (1.1–1.9)***
D dimer, mean (IQR), ng/mL	1593 (720–3790)	2260 (1009–4894)	1319 (634–3548)	1090 (580–2100)***
Ferritin, mean (IQR), ng/mL	1600 (1290–2240)	1800 (1416–2377)	1554 (1271–1936)	1538 (1280–1899)***
*Treatments*				
Corticosteroids, *n* (%)	1171 (58.0)	535 (62.4)	338 (54.3)	298 (55.5)**
Antibiotics, * n* (%)	1814 (89.9)	780 (91.0)	573 (92.0)	461 (85.8)***
Lopinavir/ritonavir, * n* (%)	1696 (84.0)	698 (81.4)	508 (81.5)	452 (84.2)
Hydroxychloroquine, * n* (%)	1861 (92.3)	805 (93.9)	566 (90.0)	490 (91.2)
Tocilizumab, * n* (%)	573 (28.4)	234 (27.3)	211 (33.9)	128 (23.8)***
Interferon β, * n* (%)	713 (35.3)	301 (35.1)	224 (36.0)	188 (35.0)
Coexisting condition and Comorbidities				
Arterial hypertension, * n* (%)	932 (46.2)	548 (63.9)	173 (27.8)	211 (39.3)***
Obesity (BMI > 30), * n* (%) ^d^	653 (32.3)	294 (34.3)	200 (32.1)	159 (29.6)
Diabetes, * n* (%)	418 (20.7)	198 (23.1)	108 (17.3)	112 (20.9)*
Coronary arterial disease, * n* (%)	124 (6.1)	48 (5.6)	41 (6.6)	35 (6.5)
COPD, * n* (%)	148 (7.3)	73 (8.5)	38 (6.1)	37 (6.9)
Chronic renal disease, * n* (%) ^e^	85 (4.2)	44 (5.1)	10 (1.6)	31 (5.8)***
Hematologic disease, * n* (%)	72 (3.5)	30 (3.5)	22 (3.5)	20 (3.7)
Asthma, * n* (%)	120 (5.9)	34 (4.0)	45 (7.2)	41 (7.6)**
HIV, * n* (%)	5 (0.2)	2 (0.2)	1 (0.2)	2 (0.4)
Pregnancy, * n* (%)	4 (0.19)	0 (0.0)	3 (0.5)	1 (0.2)
Autoimmune disease, * n* (%) ^f^	74 (3.6)	36 (4.2)	18 (2.9)	20 (3.7)
Chronic heart disease, * n* (%) ^g^	57 (2.8)	26 (3.0)	10 (1.6)	21 (3.9)
Neuromuscular disease, * n* (%)	16 (0.8)	8 (0.9)	5 (0.8)	3 (0.6)
Oxygenation and ventilator support				
Oxygen mask, * n* (%)	325 (16.1)	96 (11.2)	105 (16.9)	124 (23.1)***
High Flow nasal cannula, * n* (%)	375 (18.6)	27 (3.2)	3 (0.5)	345 (64.2)***
Non-invasive ventilation, * n* (%)	140 (6.9)	50 (5.8)	26 (4.2)	64 (11.9)***
Invasive mechanical ventilation, * n* (%)	1172 (58.1)	694 (81.0)	475 (76.2)	3 (0.6)***
PaO2/FiO2, mean (IQR)	132 (96–163)	126 (88–155)	165 (144–212)	111 (82–133)***
Complications				
Shock, * n* (%) ^h^	904 (44.8)	652 (76.1)	196 (31.5)	56 (10.4)
Acute kidney dysfunction, * n* (%) ^i^	579 (28.7)	350 (40.8)	118 (18.9)	111 (20.7)***
Myocardial dysfunction, * n* (%) ^j^	169 (8.3)	96 (11.2)	43 (6.9)	30 (5.6)***
> 2 Quadrant infiltrates in chest x-ray, * n* (%)	1327 (65.7)	573 (66.8)	413 (66.3)	341 (63.5)
ICU crude mortality, * n* (%)	657 (32.6)	389 (45.4)	159 (25.5)	109 (20.3)***

*IQR* interquartile range, *APACHE II* Acute Physiology and Chronic Health Evaluation II, *SOFA* Sequential Organ Failure Assessment, *BMI* body mass index, *COPD* Chronic obstructive pulmonary disease, *HIV* human immunodeficiency viruses, *PaO*_*2*_*/FiO*_*2*_ Partial pressure arterial oxygen/ fraction of inspired oxygen

^a^Corresponds to minimum or maximum value, as appropriate, within 12 h of ICU admission. The variables in this Table were no transformed for your comparison

^b^APACHE II score to the severity of illness, the score is obtained by adding the following components (1) 12 clinical and laboratory variables each with a score range of 0 to 4 points (APS). The APS is determined from the worst physiologic values during the initial 24 h after ICU admission, (2) age with a range 0 to 6 points and (3) Chronic health points if the patients has history of severe organ system insufficiency or is immunocompromised assign 5 points if the patients is no operative or emergency postoperative and 2 points for elective postoperative patients with a total score range of 0 to 71

^c^SOFA score corresponds to the severity of organ dysfunction, reflecting six organ systems each with a score range of 0 to 4 points (cardiovascular, hepatic, hematologic, respiratory, neurological, renal), with a total score range of 0 to 24

^d^Defined as a body mass index (calculated as weight in kilograms divided by height in meters squared) of 30 or greater

^e^Baseline eGFR < 60 on at least two consecutive values at least 12 weeks apart prior or hemodialysis

^f^Included acute leukemia, myelodysplastic syndrome and Lymphomas

^g^According to the New York Heart Association (NYHA) Functional Classification III and IV

^h^Defined as patients in whom adequate fluid resuscitation therapy are unable to restore hemodynamic stability and need any dose of vasopressor drugs

^i^Define as an abrupt and sustained (more than 24 h) decrease in kidney function and categorized according to RIFLE criteria

^j^Define as an acute decrease in ejection fraction (EF) with dilatation of ventricles observed in echocardiography upon ICU admission

All comparison between clusters. *p < .05; **p < .01; ***p < .001, others comparison *p* > .01

**Table 3:** Performance of global and clustering models (GLM model: generalized linear models with variables according ICU mortality table; Global model: GLM model with important variables according to information value analysis for ICU mortality; A, B and C Phenotypes: GLM models with important variables according to information value analysis for each cluster)

Variable	Classic model	Automatic model	A Phenotype	B phenotype	C phenotype
No. patients	2022	2017	537	623	857
Variables included	25	25	26	19	18
Accuracy	0.78	0.77	0.86	0.80	0.72
Sensitivity	0.88	0.88	0.94	0.92	0.71
Specificity	0.45	0.54	0.55	0.47	0.73
AUC ROC	0.83	0.83	0.90	0.85	0.79

**Table 4:** Factors independently associated with ICU mortality in automatic and clustering models (automatic model: generalized linear model [GLM] with important variables according to information value analysis for ICU mortality; A, B and C Phenotypes: GLM models with important variables according to information value analysis for each cluster)

Automatic model		A phenotype		B phenotype		C phenotype	
Variables^a^	OR (95%CI)	Variables^a^	OR (95%CI)	Variables^a^	OR (95%CI)	Variables^a^	OR (95%CI)
Age 56–66y	2.39 (1.68–3.41)	Age > 61y	4.37 (2.16–9.31)	Age 58–66 y	2.79 (1.40–5.76)	Age 56–73y	2.22 (1.42–3.07)
Age > 66–73y	3.51 (2.44–5.08)			Age > 66 y	5.75 (3.10–11.1)	Age 74–78y	3.79 (2.14–6.79)
Age > 73	5.97 (4.05–8.82)					Age > 78y	21.6 (6.58–99.8)
APACHE II 15–19	1.88 (1.36–2.63)	APACHE II > 14	2.21 (1.20–4.10)			APACHEII > 16	1.84 (1.21–2.80)
APACHE II > 19	1.67 (1.15–2.45)						
SOFA 3–7	1.57 (1.09–2.27)	SOFA > 4	2.20 (1.19–4.10)				
SOFA 7–8	1.66 (1.03–2.67)						
SOFA > 8	1.70 (1.05–2.77)						
COPD	1.50 (1.01–2.24)			COPD	2.30 (1.03–5.10)		
> 3 infiltrates	1.72 (1.02–1.90)					No infiltrates	0.15 (0.02–0.74)
LDH > 500 U/L	1.30 (1.02–1.66)			LDH > 800 U/L	2.66 (1.22–5.84)	LDH > 500 U/L	1.56 (1.03–2.38)
PCT > 0.45 ng/mL	1.36 (1.01–1.86)						
Lactate > 2.2 mmol/L	1.78 (1.34–2.37)			Lactate 1–1.7 mmol/L	3.13 (1.14–10.2)	Lactate > 2.0 mmol/L	1.90 (1.34–2.87)
				Lactate > 1.7 mmol/L	4.04 (1.45–13.4)		
D Dimer > 4000 ng/mL	1.44 (1.10–1.89)	D Dimer > 2000 ng/mL	2.46 (1.23–4.94)	D Dimer < 300 ng/mL	0.07 (0.01–0.33)	D Dimer > 3000 ng/mL	1.46 (1.04–2.05)
Ferritin 1700–3350 ng/mL	1.65 (1.28–2.12)	Ferritin > 1600 ng/mL	2.23 (1.154.36)			Ferritin > 3000 ng/mL	2.09 (1.06–4.21)
Ferritin > 3350	3.24 (1.82–5.84)						
PaO_2_/FiO_2_ > 140	0.65 (0.50–0.84)					PaO2/FiO2 > 150	0.60 (0.40–0.88)
GAP-UCI > 8 days	1.61 (1.00–2.60)					GAP-UCI > 8 days	3.97 (1.81–9.4)
AKI	2.53 (1.94–3.32)	AKI	3.41 (1.72–6.84)	AKI	2.11 (1.22–3.64)	AKI	2.05 (1.45–2.91)
		Hydroxychloroquine	0.33 (0.12–0.87)				
		Myocardial dysfunction	3.16 (1.14–8.78)				
						Hematologic Dis	3.01 (1.22–7.97)
						Coronary Dis	2.71 (1.25–6.27)

*OR* Odds ratio, *CI* Confidence interval, *APACHE II* Acute Physiology and Chronic Health Evaluation II, *SOFA* Sequential Organ Failure Assessment, *COPD* Chronic obstructive pulmonary disease, *LDH* D-Lactate dehydrogenase, *PCT* Procalcitonin, *GAP-UCI* Time in days from symptoms onset and ICU admission, *PaO*_*2*_*/FiO*_*2*_ Partial pressure arterial oxygen/fraction of inspired oxygen, *AKI* acute kidney injury

^a^Corresponds to categorized variables independently associated with ICU mortality

### Construction of the ICU Mortality classic multivariate model

**Fig. 4:**
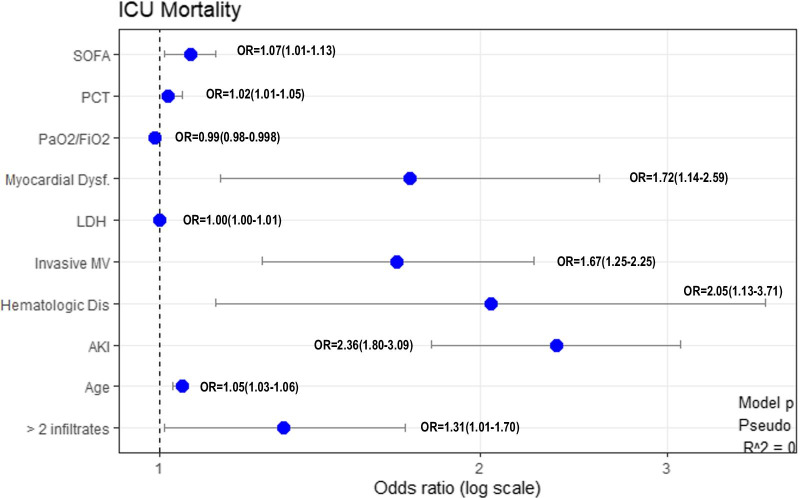
Variables independently associated with ICU mortality in multivariable analysis (GLM: generalized linear model). Data are show as OR (odds ratio) and 95% Confidence interval (*SOFA* Sequential organ failure assessment, *PCT* Procalcitonin, *PaO*_*2*_*/FiO*_*2*_ Partial pressure arterial oxygen/fraction of inspired oxygen, *Dysf* Dysfunction, *LDH* D-Lactate dehydrogenase, *MV* Mechanical ventilation, *AKI* Acute Kidney injury, > *2 infiltrates*  > 2 infiltrates in chest-X ray)

Of the 42 variables measured at ICU admission, 25 variables that were statistically significant in the univariate analysis (Table 1) were included in the model. The initial dataset of patients was randomly split in two subgroups “Training group” with 1,618 patients (80%) and “Test group” with 404 patients (20%). The characteristics of patients included into each subgroup are shown in Additional file 1: e-Table 3, p. 8. No significant differences were observed between the subgroups. Inclusion of these 25 variables in a GLM model for the training group, resulted in 10 variables that were independently associated with ICU mortality (Fig. 4). No presence of collinearity between explanatory variables was observed (Additional file 1: p. 11) and the Hosmer–Lemeshow Goodness-of-fit test (X-squared = 5.53, df = 8, p-value = 0.69) established no discrepancy between the observed values and those that would have been expected in the model. The validation of the classic model in the test group demonstrated adequate performance with an accuracy of 0.78, a precision test of 0.73, sensitivity of 0.88, specificity of 0.45 and an AUC ROC of 0.82 (95%CI 0.78–0.86) (Additional file 1: p. 10). Performance of classic model was similar than automatic model (Table 3), however, the variables included in each model were different (Fig. 4 and Additional file 1: e-Fig. 9, p. 16).

## Discussion

The main finding of our study is that among patients with COVID-19, 3 clinical phenotypes were derived using habitual clinical and laboratory variables at ICU admission. The ability of identifying phenotypes using a small set of variables is a crucial step towards clinical application and has important implications for possible differential treatment guided by phenotypes and validated prognostic scoring systems [18, 19].

Our C phenotype was associated with more than double the ICU mortality than each of the remaining two phenotypes. This C phenotype was characterized by the interplay of older age (> 65 year), a high severity (APACHE II > 15 and SOFA > 5), greater burden of risk factors (hematologic disease and coronary disease) and a higher likelihood of developing further complications (shock and AKI).

Previous studies have implemented clustering techniques to analyze various data sources relating to demographic, geographic, environment, and socioeconomic determinants of health and disease. There are studies that have evaluated treatment decisions and characterized clinical phenotypes associated with complications, ICU admission and mortality risk in critically ill COVID-19 patients. According to the Situation Report & Public Health Guidance published by Johns Hopkins University on March 19th, 2020, people over 60 and those with chronic health conditions are at the highest risk for COVID-19 complications [20]. To our knowledge, this is the first study with a high number of critically ill patients to analyze the presence of phenotypes in patients with SARS-CoV-2 infection. This multicenter cohort study of 2,022 critically ill patients found that 660 patients (32.6%) died at ICU discharge. Our ICU mortality rates was significantly lower as reported in Yang et al. [4] in Wuhan, China (61.5%), by Myers et al. [7] in California, USA (50.0%), by Arentz et al. [6] in Washington, USA (67%) and by Richardson et al. [5] in New York, USA (78%), but slightly higher to reported by Grasselli et al. [8] in Lombardy region, Italy (26%). These observed differences in ICU mortality could respond to different healthcare models and important practice variations in the management of severe COVID-19 patients [11], but it can also depend on the frequency of presentation of the different phenotypes.

In our study, a great variability in model performance and risk factors were observed during cross-validation to choose the best model to use. In addition, we use 2 different techniques for the selection of important variables, one of them is the “classic” approach dependent on the p-value, while the other, a “modern” statistical approaches, is more in line with the new recommendations [21]. Although the performance of the models was similar, the variables included in each of them are different. This could be related to the presence of a very heterogeneous patient population, which is revealed during random partitioning (80%/20%) validation of each model or by implementing 2 variable selection techniques. In this context, three clinical phenotypes of COVID-19 patients were derived using routinely available clinical data at ICU admission by an unsupervised cluster analysis. The phenotypes were multidimensional, differed in their demographics, laboratory abnormalities, patterns of organ dysfunction, and associated with ICU mortality. In addition, our phenotypes are not similar with groupings or phenotypes of patients performed so far considering only the presence of clinical complications [22, 23], or the type of ARDS [24]. Our COVID-19 phenotypes can be identified at the time of the ICU admission, and thus could be useful in facilitating early tailored therapy and improve prognosis.

Only routinely available clinical and laboratory data were used in the clustering models, and the phenotypes were derived from a large observational multicenter cohort to ensure generalizability. Importantly, we have observed that the variables associated with the ICU mortality varied between the global model and the models developed for each phenotype. The discrimination power (AUC) of A and B phenotypes models improved in comparison to the global model. However, for the C phenotype (severe COVID-19 disease), the performance of the model was not superior respect of the global model. The C phenotype was most strongly correlated with abnormal values of biomarkers as well as clinical features of cardiovascular dysfunction, AKI and subsequently a higher ICU mortality. Although the AUC for C phenotype is lower, the relationship between sensitivity and specificity in C phenotype model might be more appropriate. Specificity can sometimes be more important than sensitivity, because confirming that a person does not have the event under study (survival) is more important than detecting if a person has it.

Recently, several authors have proposed different clinical phenotypes of COVID-19 patients [22–24]. Rello et al. [22] speculated that COVID-19 has five phenotypic presentations based on physiological and clinical features from published studies. Garcia-Vidal et al. [23] describe the main clinical complications of hospitalized patients with COVID-19 through classification into three pattern groups (inflammatory, co-infection and thrombotic). However, as the authors acknowledge, the cut-off points of the different biomarkers for defining phenotypes have been arbitrary and not scientifically supported. Finally, Gattinoni et al. [24] proposing two phenotypes for COVID-19 patients, (1) “Type L” characterized by high compliance and low lung recruitablity and (2) “Type H” with low compliance and high lung recruitability as a two “extremes” of a spectrum of respiratory failure in COVID-19 pneumonia. Despite the importance related to clinical experience in each of these approaches, none of these studies have been developed through a machine learning process to determine phenotypes nor have they been tested for validation.

Hypoxemia has been proposed as a marker of severity for the differentiation of phenotypes [22, 24]. In our study, the PaO_2_/FiO_2_ relationship at ICU admission was an independent risk factor for ICU mortality in overall multivariate analysis (as a continuous or dichotomized variable), but was only closely associated with ICU mortality in phenotype C. Other variables such as advanced age, serum D-dimer values and the development of AKI were variables more strongly related to ICU mortality in all subgroups or phenotypes analyzed than PaO_2_/FiO_2_ at ICU admission.

Our results should be interpreted in the context of the study limitations. First, although phenotypes were found to be generalizable in our population, risk factors and characteristics of clinical phenotypes were derived initially from data at ICU admission of multicenter observational study in Spain. However, these risk factors are similar to those that have been reported by other investigators [4–8]. The cross-validation carried out and the high discrimination observed for each of the models built for phenotypes, suggests their applicability to other populations, but it should be examined considering the high variability observed in patients with COVID-19 and in the support measures applied. Second, because missing data were common for some variables included in the clustering models, multiple imputation was used in the primary analysis. However, variables with high missing values were excluded and the missing threshold used was reported elsewhere [18]. Third, only routinely available clinical data at ICU admission were used to identify risk factors and clinical phenotypes, and the inclusion of other data related to clinical evolution of patients in the ICU could change risk factors or phenotype assignments. However, our objective was to study early risk factors and phenotypes at ICU admission that may allow for early treatment implementation and as a result improve patient outcome. Fourth, although IL-6 is an excellent severity biomarker, we have not been able to include this biomarker in the models because more than 50% of patients had no IL-6 determination upon ICU admission. Although the inclusion of IL-6 in models could modify or improve their performance, we do not consider it appropriate to impute a large number of missing data. In addition, if IL-6 is a biomarker not usually available its inclusion in the models would not have practical application. Finally, we did not collect data on ethnicity or socioeconomic factors. These factors may play a role in the prevalence of pre-existing comorbidities and mortality due to COVID-19. Our findings should be interpreted within the context of the study population and its generalizability to other populations warrant further investigation.

## Conclusion

To our knowledge this is the largest study that describe different phenotypes of patients with confirmed COVID-19 that were admitted to ICU to date. We not only characterized three novel clinical phenotypes, but extended findings outside of a single site ICU by characterizing the association of comorbidities with clinical phenotype and the association of clinical phenotypes with clinical outcomes. Different risk factors for the global population and clinical phenotypes were observed, possibly due to the heterogeneity of patients, which may limit the application of a single predictive model for all patients with COVID-19. Further research is needed to determine the application of these phenotypes in clinical practice, in other patient’s population and for clinical trial design.

## Supplementary Information

**Additional file 1**. Supplementary online content.

## Data Availability

The anonymized database collected for the study by the SEMICYUC, and the data dictionary that defines each field in the set, will be made available to reviewers if they consider it necessary prior confidentiality agreement.

## Abbreviations

ADRS: Acute distress respiratory syndrome
AKI: Acute kidney injury
APACHE II: Acute Physiology and Chronic Health Evaluation II
AUC: Area Under the ROC Curve
CKD: Chronic kidney disease
CI: Confidence interval
COPD: Chronic obstructive pulmonary disease
CRP: C-Reactive Protein
GLM: Generalized linear regression model
HFNC: High flow nasal cannula oxygen therapy
ICC: Interclass correlation coefficient
ICU: Intensive Care Unit
IQR: Interquartile range
IV: Information value
LDH: D-lactate dehydrogenase
LOS: Length of stay
MV: Mechanical ventilation
PAM: Partition around medoids
PCA: Principal component analysis
PCT: Procalcitonin
ROC: Receiver Operator Characteristic curve
RT-PCR: Real time polymerase chain reaction
SEMICYUC: Sociedad Española de Medicina Intensiva, Critica y Unidades Coronarias
SOFA: Sequential Organ Failure Assessment
VIF: Variance inflation factors
WHO: World Health Organization
