# Supplementary material for: Deploying unsupervised clustering analysis to derive clinical phenotypes and risk factors associated with mortality risk in 2022 critically ill patients with COVID-19 in Spain
Source: Crit Care. 2021 Feb 15;25:63. doi: 10.1186/s13054-021-03487-8 (PMC7883885; doi:10.1186/s13054-021-03487-8)

**Additional file R1**

**Deploying unsupervised clustering analysis to derive clinical phenotypes and risk factors associated with mortality risk in 2,022 critically ill patients with COVID-19 in Spain Supplementary Online Content**

**e-Methods**. Supplemental Methods

**e-Figure 1:** Heat map of correlation between clinical variables for analysis and phenotyping

**e-Table 1**. Definitions of Baseline Characteristics, Comorbidities, Treatments, and Outcomes

**e-Figure 2.** The Random-effects plot for hospital

**e-Table 2.** Crude ICU Mortality according to predefined age cut-off.

**e-Figure 3.** Crude ICU mortality plot according to age

**e-Table 3.** Characteristics of 2022 patients according to outcome

**e-Figure 4.** ROC curve plot for the GLM ICU mortality model

**e-Table 4.** Variance Inflation Factors (VIF)

**e-Table 5.** Ranking of variables according to the information value(IV)

**e-Figure 5.** Categorized variables independently associated with ICU mortality

**e-Figure 6.** Silhouette plot

**e-Figure 7.** PAM Plot

**e-Figure 8.** Plot of clinical phenotypes

**e-Table 6.** Ranking of variables according to the iv in each phenotype.

**e-Figure 9.** Variables independently associated with ICU mortality rate in global model and in each phenotype

**eMethods: Supplemental Methods**

**Data Collection and Validation**

Data were collected using a paper CRF (case Report Form). CRF collect and record all protocol-required information, which is transcribed from patient source documents, such as hospital records and laboratory reports during the patient's participation in the study. Before being sent to the Study Coordinator (AR) this data was de-identified (not traceable to the patient) by removing the patient's name, medical record number, etc., and giving the patient a unique study number. We implemented a double data entry model for potential errors in real-time. Data was entered twice by two different Data Entry personnel based on the same set of data collected in the paper CRFs. All data were reviewed, and values that appeared incongruent or out of range were manually validated by confirming the accuracy of the data with the Study Coordinator (AR). The database was validated and cleaned before the statistical analysis and finally, the study database was locked to prevent any further changes, and to ensure data consistency and integrity for the statistical reporting and analysis.

**Approach to missing data**

Continuous variables with missing data > 30% were excluded of database. Missing data for continuous variables were imputed using R-package "missForest" for the statistical software R/CRAN. The imputation was applied to impute the missing values of D dimer (20%), Ferritin (20%), D-Lactate dehydrogenase (17%), Procalcitonin (17%), creatinine (16%), SOFA score (16%), APACHE II score (10%) and C-reactive protein (CRP) (5%). Categorical data (including ICU mortality) were available for all patients.

**Study definitions**

Community-acquired pneumonia (CAP) was defined in accordance with current American Thoracic Society and Infectious Diseases Society of America guidelines (ATS/IDSA )(1).

Primary viral pneumonia due to SARS-CoV-2 infection was defined by the presence of acute respiratory failure and unequivocal alveolar opacities involving one or more lobes, with negative respiratory and blood bacterial cultures at ICU admission.

Community-acquired respiratory co-infection (CARC) was considered in patients with confirmation of SARS-CoV-2 infection showing recurrence of fever, increase in cough and production of purulent sputum plus positive bacterial/fungal respiratory or blood cultures at ICU admission (2).

Shock was defined in accordance with the Surviving Sepsis Campaign guidelines (3); that is, patients in whom adequate fluid resuscitation and vasopressor therapy are unable to restore hemodynamic stability.

Acute Kidney injury (AKI) was defined according to Consensus Conference of the Acute Dialysis Quality Initiative (4).

Acute respiratory distress syndrome (ARDS) was defined according Berlin definition (5) in 3 categories based on degree of hypoxemia: mild (PaO2/FIO2 ≤ 300 mm Hg), moderate (PaO2/FIO2 ≤ 200 mm Hg), and severe (PaO2/FIO2 ≤ 100 mm Hg)

**Statistical analysis**

To performed multivariate model and to derive the phenotypes, we first assessed the candidate variables, missing data and correlation by Cramer’s V. After evaluating correlation, highly correlated were excluded (**eFigure1**).

To determine if a significant inter-hospital variation is present, multilevel conditional logistic modelling with patients nested in hospital to characterize hospital-level variation of ICU mortality was done. We built an empty model, to assess the variation of the log-odds from one hospital to another and calculate the intraclass correlation coefficient (ICC) for one-way random-effects model. The ICC quantifies the degree of homogeneity of the outcome within clusters and represents the proportion of the between-hospital variation in the total variation. When the ICC is not different from zero or negligible, indicates perfect independence of residuals and traditional one level regression analysis can be done (6). The ICC obtained when considering all hospital (n=63) was 0.04 **(eFigure 2)** and was no different when excluded hospitals that submitted data on few than 10 patients (ICC=0.04, data no shown). This ICC represents that the hospital-level variation was very poor (4%) and a one-level regression analysis can be used.

Mod1 <- glmer(deadUCI ~ ( 1 | hospital), data=df, family = "binomial")

summary(Mod1)

Generalized linear mixed model fit by maximum likelihood (Laplace Approximation) [glmerMod]

Family: binomial ( logit )

Formula: deadUCI ~ (1 | hospital)

Data: df

AIC BIC logLik deviance df.resid

2537.4 2548.6 -1266.7 2533.4 2020

Scaled residuals:

Min 1Q Median 3Q Max

-0.9513 -0.6949 -0.6204 1.2060 1.9144

Random effects:

Groups Name Variance Std.Dev.

hospital (Intercept) 0.1492 0.3862

Number of obs: 2022, groups: hospital, 63

Fixed effects:

Estimate Std. Error z value Pr(>|z|)

(Intercept) -0.74458 0.07417 -10.04 <2e-16 ***

---

Signif. codes: 0 ‘***’ 0.001 ‘**’ 0.01 ‘*’ 0.05 ‘.’ 0.1 ‘ ’ 1

Intraclass correlation coefficient

icc <- Mod1@theta[1]^2/ (Mod1@theta[1]^2 + (3.14159^2/3))

icc

[1] 0.04337433

To investigate the association between baseline (on ICU admission) variables and ICU-mortality, a multivariate analysis (GLM: Generalized linear Regression model) in R statistical software was performed (Stats package https://www.rdocumentation.org/packages/stats). The multivariate model comprised factors of clinical interest and all significant covariates (p<0.05) in the univariate analysis of ICU mortality. GLM in R is a class of regression models that supports non-normal distributions, and can be implemented in R through glm() function that takes various parameters and the model works well with a variable which depicts a non-constant variance.

We studied presence of collinearity between explanatory variables with the use of variance inflation factors (VIF). A VIF value greater than 5 was considered as potentially severe correlation between predictor variables and the variable was excluded of model.

Finally, and to determine presence of clinical phenotypes an unsupervised clustering analysis was applied to the database at ICU admission. In order to carry out this analysis, a discretization of the numerical variables into categorical ones was done. The information provided by each variable regarding ICU mortality was defined using the Information Value (IF).

IV is one of the most useful technique to select important variables in a predictive model. It helps to rank variables on the basis of their importance. The IV was calculated using the following formula:

IV = ∑ (% of non-events - % of events) * WOE#

#The weight of evidence (WOE) tells the predictive power of an independent variable in relation to the dependent variable.

According to Sidoqqui recommendation (7) an IF greater than 0.03 was considered clinically important and this variable was included in the GLM analysis. Model performance was examined using accuracy test, Sensibility, Specificity and AUC. Subsequently, the unsupervised cluster analysis was performed using the important variables.

The Podani distance was used to calculate the distance between patients and the “partition around medoids” (PAM) algorithm to perform the clustering (8). The algorithm is intended to find a sequence of objects called medoids that are centrally located in clusters. Objects that are tentatively defined as medoids are placed into a set S of selected objects. If O is the set of objects that the set U = O − S is the set of unselected objects. The goal of the algorithm is to minimize the average dissimilarity of objects to their closest selected object. It is more robust to noise and outliers as compared to k-means because it minimizes a sum of pairwise dissimilarities instead of a sum of squared Euclidean distances. The optimal number of clusters were determined after studying the silhouette (9) and the PAM objective for different numbers of clusters. Each of these clusters represent a specific patient’s phenotype.

**Circularity Bias approach during model selection and evaluation**

The term "circular analysis" or “circularity bias” covers a wide variety of situations and their can appear for example, with the use of the same data to train and test a classifier (10). To avoid the circularity bias, in our analysis a cross-validation (80%+20%) K-Fold= 10 was made to obtain the classic model. On the other hand, to develop the automatic model, the variables to include were selected by automatic methods and subsequently a validation of the model was performed with a subset of train and test.

Finally, circularity bias can arise when the data are first analyzed to select a subset, and then the subset is reanalyzed to obtain the results. In this context, assumptions and hypotheses determine the selection criterion, and selection, in turn, can distort the results (11). Our analysis to derivation of phenotypes was performed by an unsupervised analysis and each model was validated with a subset of train and test. This prevents the presence of circulatory bias.

References

1.- Metlay JP,Waterer GW,Long AC, Anzueto A, Brozek J, Crothers K, et al. Diagnosis and Treatment of Adults with Community-acquired Pneumonia. An Official Clinical Practice Guideline of the American Thoracic Society and Infectious Diseases Society of America. Am J Resp Crit Care Med 2019 ;200(7): e45-e67. https://www.atsjournals.org/doi/full/10.1164/rccm.201908-1581ST

2.- Martin-Loeches I, Schultz MJ, Vincent JL, Alvarez-Lerma F, Bos LD, Sole-Violan J et al. Increased incidence of co-infection in critically ill patients with influenza. Intensive Care Med 2017; 43:48–58. https://www/doi/10.1007/s00134-016-4578-y

3.-Dellinger RP, Levy MM, Rhodes A, Annane D, Gerlach H, Opal SM et al. Surviving sepsis campaign: international guidelines for management of severe sepsis and septic shock: 2012. Crit Care Med 2013;41(2):580-637. https://www/doi/10.1097/CCM.0b013e31827e83af.

4.- Bellomo R. Ronco C. Kellum J.A. et al. Acute renal failure—definition, outcome measures, animal models, fluid therapy and information technology needs: the Second International Consensus Conference of the Acute Dialysis Quality Initiative (ADQI) Group. Crit Care. 2004; 8: R204-R212

5.- Ranieri VM, Rubenfeld GD, Thompson BT, Ferguson ND, Caldwell E, Fan E, Camporota L, Slutsky AS. Acute respiratory distress syndrome: The Berlin Definition ARDS Definition Task Force. JAMA 2012;307(23):2526-33. https://doi/10.1001/jama.2012.5669.

6.- Sommet, N. and Morselli, D. (2017). Keep Calm and Learn Multilevel Logistic Modeling: A Simplified Three-Step Procedure Using Stata, R, Mplus, and SPSS. International Review of Social Psychology, 30(1), 203–218, DOI: https://doi.org/10.5334/irsp.90.

7.- Siddoqi N. Scorecard Development Process. En: Siddoqi N, editor. Credit Risk Scorecards: Developing and Implementing Intelligent Credit Scoring. New Jersey: John Wiley & Sons; 2015. p. 73---127.

8.- Van der Laan M, Pollard K, Bryan J. A new partitioning around medoids algorithm, Journal of Statistical Computation and Simulation 2003; 73:8, 575-584. https://www/doi/10.1080/0094965031000136012

9.-Rousseeuw, P.J. (1987) Silhouettes: A graphical aid to the interpretation and validation of cluster analysis. J. Comput. Appl. Math. 1987; 20:53–65 https://doi.org/10.1016/0377-0427(87)90125-7

10.- Kriegeskorte N, Lindquist MA, E Nichols TE et al. Everything you never wanted to know about circular analysis, but were afraid to ask. Journal of Cerebral Blood Flow & Metabolism 2010; 30, 1551–1557

11.- Kriegeskorte N, Simmons WK, Bellgowan PSF, Baker CI. Circular analysis in systems neuroscience – the dangers of double dipping. Nat Neurosci. 2009; 12(5): 535–540.

**eFigure 1: Heatmap of correlation between clinical variables for analysis and phenotyping**

**
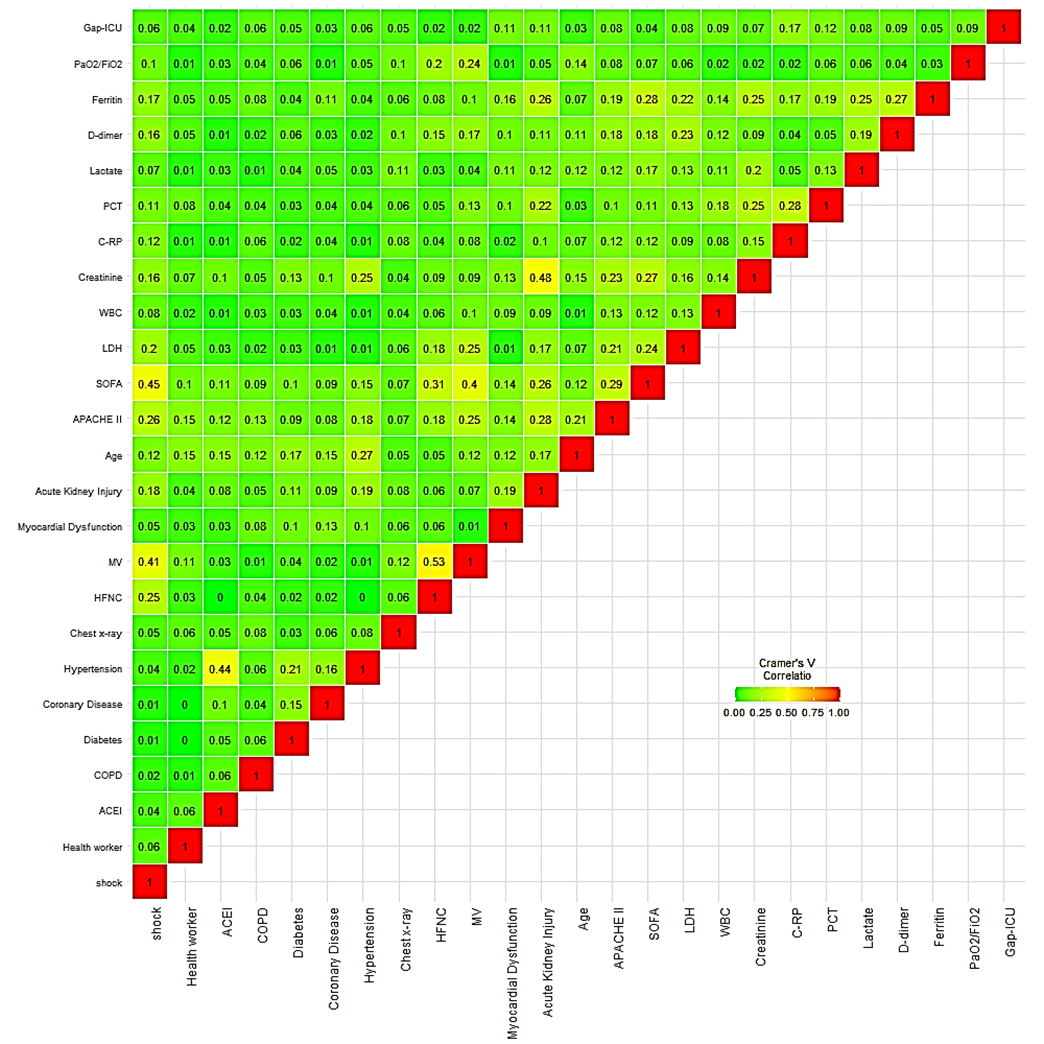
**

**eTable 1**: Definitions of variables recorded, Comorbidities, Treatments, and Outcomes Collected in patients

| **General characteristics and severity of illness** | |
| --- | --- |
| Hospital type | According to beds number (<200 ; 200-500 and > 500 beds) |
| Sex | 1:Male; 0: Female |
| Age | Number of years of age at the time of ICU admission |
| Date of Hospital admission | Per chart review |
| Data of ICU admission | Per chart review |
| Data of ICU discharge | Per chart review |
| Data of Hospital discharge | Per chart review |
| GAP ICU | Time in days from Hospital to ICU admission |
| GAP diagnosis | Time in days from onset of symptoms to diagnosis |
| GAP antiviral treatment | Time in days from onset of symptoms to first dose of antiviral |
| APACHE II score | Acute Physiology and Chronic Health Evaluation (APACHE) II score was calculated for all patients within the first 24 h of ICU admission |
| SOFA score | Sequential Organ Failure Assessment (SOFA) scoring system was calculated for all patients within the first 24 h of ICU admission. |
| Health worker | People who work in the hospital or other areas of health care |
| **Comorbidities and coexisting conditions** | |
| 9.- Asthma | Per chart review |
| 10.- Chronic Pulmonary Obstructive Disease | Per chart review |
| 11.- Arterial Hypertension | Per chart review |
| 12.- Chronic Heart disease | Per chart review. New York Heart Association (NYHA) Functional Classification III and IV |
| 13.- Chronic kidney disease | Baseline eGFR< 60 on at least two consecutive values at least 12 weeks apart prior or hemodialysis |
| 14.- Hematologic disease | Per chart review, included acute leukemia, myelodysplastic syndrome and Lymphomas. |
| 15.- Pregnancy | Per chart review |
| 16.- Obesity | Body mass index > 30 |
| 17.- Diabetes mellitus | Per chart review |
| 18.- HIV/AIDS | Per chart review |
| 19.- Coronary artery disease | Per chart review |
| 20.- Neuromuscular disease | Per chart review |
| 21.- Immunological disease | Per chart review |
| 22.- Other Immunodeficiency disorders | Per chart review |
| **Laboratory findings** | |
| D-Lactate dehydrogenase | U/L, Per laboratory report |
| White blood cell | x10^9^ Per laboratory report |
| Serum Creatinine | mg/dL, Per laboratory report |
| C-Reactive Protein (CRP) | mg/mL, Per laboratory report |
| Procalcitonin (PCT) | ng/mL, Per laboratory report |
| Serum lactate | mmol/L, Per laboratory report |
| D dimer | ng/mL, Per laboratory report |
| Ferritin | ng/mL, Per laboratory report |
| Arterial blood gas (ABG) test | Per laboratory report |
| **Treatment at ICU admission** | |
| Corticosteroids | Per chart review |
| Antibiotics | Per chart review |
| Lopinavir/ritonavir | Per chart review |
| Hydroxychloroquine | Per chart review |
| Tocilizumab | Per chart review |
| Interferon β | Per chart review |
| Corticosteroids | Per chart review |
| Antibiotics | Per chart review |
| Lopinavir/ritonavir | Per chart review |
| Anti-hypertensive treatment | ACEI: Angiotensin Converting Enzyme Inhibitors; ARB: Angiotensin receptor blockers |
| **Oxygenation and ventilator support at ICU admission and at first 24 hours** | |
| Oxygen mask | Per chart review |
| High Flow nasal cannula | Per chart review |
| Non-invasive ventilation | Per chart review |
| Invasive mechanical ventilation | Per chart review |
| Oxygen mask | Per chart review |
| High Flow nasal cannula | Per chart review |
| **Complications at ICU admission** | |
| Shock | Per chat review |
| Acute kidney dysfunction | Per chat review |
| Myocardial dysfunction | Per chat review |
| Community-acquired co-infection | Per chat review |
| > 2 Quadrant infiltrates in chest x-ray | Per chest x-ray review |
| **Outcome** | |
| ICU crude mortality | Per chart review |

**eFigure 2**: Random-effects plot for hospital. The intercept or average math score for each hospital (ID). The random-effects was obtained using the function ranef().

bi<-ranef(Mod1)

hosp.intercept <- bi$hospital + -0.74458

plot(hosp.intercept[,1],type='h',

xlab='hospital ID', ylab='Intercept')

abline(h=-0.74458)


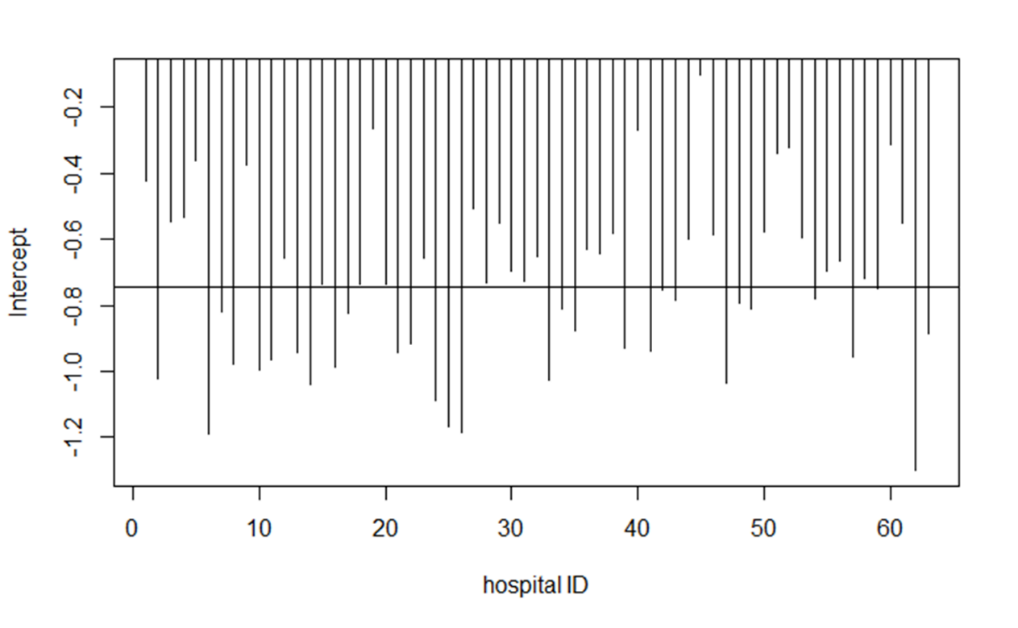


**eTable 2**: Crude ICU Mortality according to predefined age cut-off

| Age cut-off | N | Nonsurvivors (%) | p-value |
| --- | --- | --- | --- |
| 16-30 | 24 | 5(17.2) | 0.01 |
| 31-40 | 74 | 11(14.9) |  |
| 41-50 | 208 | 21(10.0) |  |
| 51-60 | 457 | 91(19.9) |  |
| 61-70 | 678 | 232(34.2) |  |
| 71-80 | 539 | 270(50.0) |  |
| >80 | 37 | 30(81.0) |  |

**eFigure 3**: Crude ICU mortality plot according to age


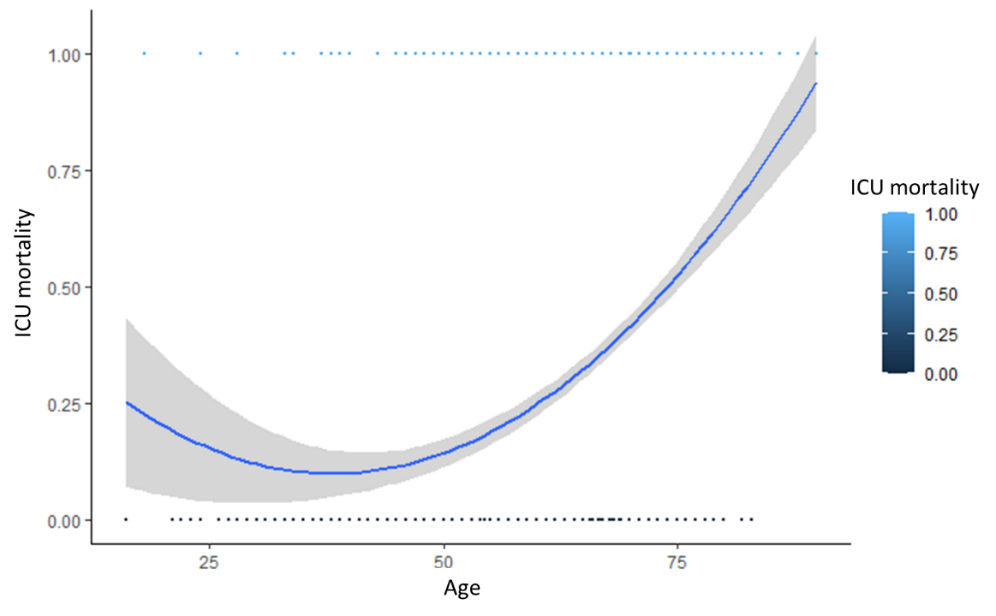


**e-Table 3**: Characteristics of 2022 critically ill patients included in the study at ICU admission according to train or test population

| Variable^a,l^ | Overall | Train population | Test population | p-value |
| --- | --- | --- | --- | --- |
| General characteristics and severity of illness | | | |  |
| No. of patients(%) | 2022 (100.0) | 1618 (80.0) | 404 (20.0) | ----- |
| Age ,median (IQR), y | 64(55-71) | 64(55-71) | 65(56-72) | 0.55 |
| Age >60 years, No.(%) | 1254(62.0) | 1006(62.2) | 248(61.4) | 0.81 |
| Male, No.(%) | 1423(70.4) | 1129(69.7) | 294(72.7) | 0.26 |
| APACHE II , median (IQR)^b^ | 13(10-17) | 14(11-18) | 15(11-18) | 0.59 |
| SOFA, median (IQR)^c^ | 5.0(3-7) | 5.6(4.0-7.7) | 5.2(4.0-7.0) | 0.10 |
| Laboratory findings | | | |  |
| D-Lactate dehydrogenase, median (IQR), U/L | 537(417-707) | 535(410-690) | 528(417-680) | 0.92 |
| White blood cell, median (IQR), x10^9^ | 8.8(6.2-12.2) | 8.8(6.2-12.3) | 9.0(6.2-11.7) | 0.98 |
| Serum Creatinine , median (IQR), mg/dL | 0.8(0.7-1.1) | 0.8(0.7-1.1) | 0.8(0.7-1.1) | 0.36 |
| C-Reactive Protein, median (IQR), mg/mL | 15.5(9.1-24.3) | 15.6(9.3-23.8) | 15.7(8.6-24.5) | 0.15 |
| Procalcitonin, median (IQR), ng/mL | 0.3(0.1-2.0) | 0.3(0.1-0.8) | 0.3(0.1-0.8) | 0.83 |
| Serum lactate, median (IQR), mmol/L | 1.5(1.1-2.0) | 1.5(1.1-2.0) | 1.6(1.2-2.0) | 0.09 |
| D dimer, median (IQR), ng/mL | 1593(720-3790) | 1570(735-3390) | 1515(680-3380) | 0.97 |
| Ferritin ng/mL, median (IQR), | 1617(1290-2240) | 1481(1200-1780) | 1476(1252-1740) | 0.66 |
| Treatments | | | |  |
| Corticosteroids, No.(%) | 1174(58.1) | 942(58.2) | 232(57.4) | 0.67 |
| Antibiotics , No.(%) | 1818(89.9) | 1452(89.7) | 366(90.6) | 0.57 |
| Lopinavir/ritonavir , No.(%) | 1662(82.2) | 1321(81.6) | 341(84.4) | 0.22 |
| Hydroxychloroquine, No.(%) | 1866(92.3) | 1492(92.2) | 374(92.5) | 0.88 |
| Tocilizumab , No.(%) | 577(28.5) | 477(29.5) | 100(24.7) | 0.06 |
| Interferon β, No.(%) | 715(35.4) | 568(35.1) | 174(43.0) | 0.67 |
| Coexisting condition and Comorbidities | | | |  |
| Arterial hypertension, No.(%) | 936(46.3) | 752(46.5) | 184(45.5) | 0.73 |
| Obesity , No.(%)^d^ | 655(32.4) | 527(32.5) | 128(31.7) | 0.73 |
| Diabetes, No.(%) | 420(20.8) | 339(20.9) | 81(20.1) | 0.74 |
| Coronary arterial disease, No.(%) | 124( 6.1) | 96(5.9) | 28(6.9) | 0.52 |
| COPD , No.(%) | 148(7.3) | 111(6.8) | 37(9.1) | 0.13 |
| Chronic renal disease , No.(%)^e^ | 85(4.2) | 71(4.4) | 14(3.5) | 0.49 |
| Hematologic disease , No.(%)^f^ | 73(3.6) | 62(3.8) | 11(2.7) | 0.35 |
| Asthma, No.(%) | 121(5.9) | 98(6.0) | 23(5.7) | 0.87 |
| HIV, No.(%) | 5(2.0) | 4(0.2) | 1(0.2) | 0.54 |
| Pregnancy , No.(%) | 4(0.2) | 3(0.18) | 1(0.2) | 1.0 |
| Autoimmune disease, No.(%) | 74(3.7) | 56(3.5) | 18(4.4) | 0.42 |
| Chronic heart disease , No.(%)^g^ | 57(2.8) | 46(2.8) | 11(2.7) | 1.0 |
| Neuromuscular disease , No.(%) | 16(0.8) | 12(0.7) | 4(1.0) | 0.89 |
| Other immunosuppression , No.(%)^h^ | 53(2.6) | 46(2.8) | 7(1.7) | 0.28 |
| Oxygenation and ventilator support | | | |  |
| Oxygen mask , No.(%) | 333(6.4) | 267(16.5) | 66(16.3) | 0.87 |
| High Flow nasal cannula, No.(%) | 375(18.5) | 298(18.4) | 77(19.1) | 0.82 |
| Non-invasive ventilation , No.(%) | 140(6.9) | 107(6.6) | 33(8.1) | 0.32 |
| Invasive mechanical ventilation, No.(%) | 1174(58.0) | 941(58.1) | 233(57.6) | 0.99 |
| Complications | | | |  |
| Shock , No.(%)^i^ | 906(44.8) | 729(45.0) | 177(43.8) | 0.69 |
| Acute kidney dysfunction , No.(%)^j^ | 580(28.7) | 477(29.5) | 103(25.5) | 0.12 |
| Community-acquired co-infection ^k^, No.(%) | 190(9.4) | 149(9.2) | 41(10.1) | 0.62 |
| > 2 Quadrant infiltrates in chest x-ray , No.(%) | 1333(65.9) | 1062(65.6) | 271(67.0) | 0.59 |
| ICU crude mortality , No.(%) | 660(32.6) | 528(32.6) | 132(32.6) | 1.0 |

Abbreviations: IQR, interquartile range; APACHE II, Acute Physiology and Chronic Health Evaluation II; SOFA, Sequential Organ Failure Assessment; BMI, body mass index; COPD, Chronic obstructive pulmonary disease; HIV, human immunodeficiency viruses; PaO2/FiO2, Partial pressure arterial oxygen/ fraction of inspired oxygen

^a^ Corresponds to minimum or maximum value, as appropriate, within 12 hours of ICU admission. The variables in this Table were no transformed for your comparison.

^b^ APACHE II score to the severity of illness, the score is obtained by adding the following components 1) 12 clinical and laboratory variables each with a score range of 0 to 4 points (APS). The APS is determined from the worst physiologic values during the initial 24 h after ICU admission, 2) age with a range 0 to 6 points and 3) Chronic health points if the patients has history of severe organ system insufficiency or is immunocompromised assign 5 points if the patients is no operative or emergency postoperative and 2 points for elective postoperative patients with a total score range of 0 to 71.

^c^ SOFA score corresponds to the severity of organ dysfunction, reflecting six organ systems each with a score range of 0 to 4 points (cardiovascular, hepatic, hematologic, respiratory, neurological, renal), with a total score range of 0 to 24,

^d^ Defined as a body mass index (calculated as weight in kilograms divided by height in meters squared) of 30 or greater.

^e^ Baseline eGFR< 60 on at least two consecutive values at least 12 weeks apart prior or hemodialysis

^f^ included acute leukemia, myelodysplastic syndrome and Lymphomas.

^g^ According to the New York Heart Association (NYHA) Functional Classification III and IV

^h^ included Chronic corticosteroid treatment (>20 mg prednisolone /day or equivalent dose), chemotherapy or therapy with biological agents

^I^ Defined as patients in whom adequate fluid resuscitation therapy are unable to restore hemodynamic stability and need any dose of vasopressor drugs.

^j^ Define as an abrupt and sustained (more than 24 hours) decrease in kidney function and categorized according to RIFLE criteria

^k^ Was considered in patients with confirmation of SARS-CoV-2 infection showing recurrence of fever, increase in cough and production of purulent sputum plus positive bacterial/fungal respiratory or blood cultures at ICU admission

^l^ Kruskal-Wallis, ANOVA, or chi-square p-value as appropriate comparing train and test population, p>.01 for all comparison

**e-Table 4**: Variance Inflation Factors (VIF) to determine presence of collinearity between explanatory variables included in the training model

| Variable | VIF |
| --- | --- |
| Age , years | 1.165966 |
| Male | 1.131517 |
| APACHE II | 1.341185 |
| SOFA | 1.577309 |
| D-Lactate dehydrogenase, U/L | 1.156532 |
| White blood cell x10^9^ | 1.129158 |
| Serum Creatinine mg/dL | 1.455142 |
| C-Reactive Protein mg/mL | 1.074133 |
| Procalcitonin ng/mL | 1.140735 |
| Serum lactate mmol/L | 1.038744 |
| D dimer ng/mL | 1.137709 |
| Ferritin ng/mL | 1.288754 |
| Arterial hypertension | 1.157518 |
| Diabetes | 1.090715 |
| Coronary arterial disease | 1.095681 |
| COPD | 1.057170 |
| Chronic renal disease | 1.186092 |
| Hematologic disease | 1.036348 |
| Other immunosuppression | 1.049225 |
| PaO2/FiO2 at ICU admission | 1.115001 |
| Invasive mechanical ventilation | 1.384719 |
| Shock | 1.378355 |
| Acute kidney dysfunction | 1.169113 |
| > 2 Quadrant infiltrates in chest x-ray | 1.048113 |
| Myocardial dysfunction | 1.080125 |

**eFigure 4:** ROC curve plot for the classic ICU mortality model. AUC represents the degree or measure of discrimination of the model for distinguishing between classes.


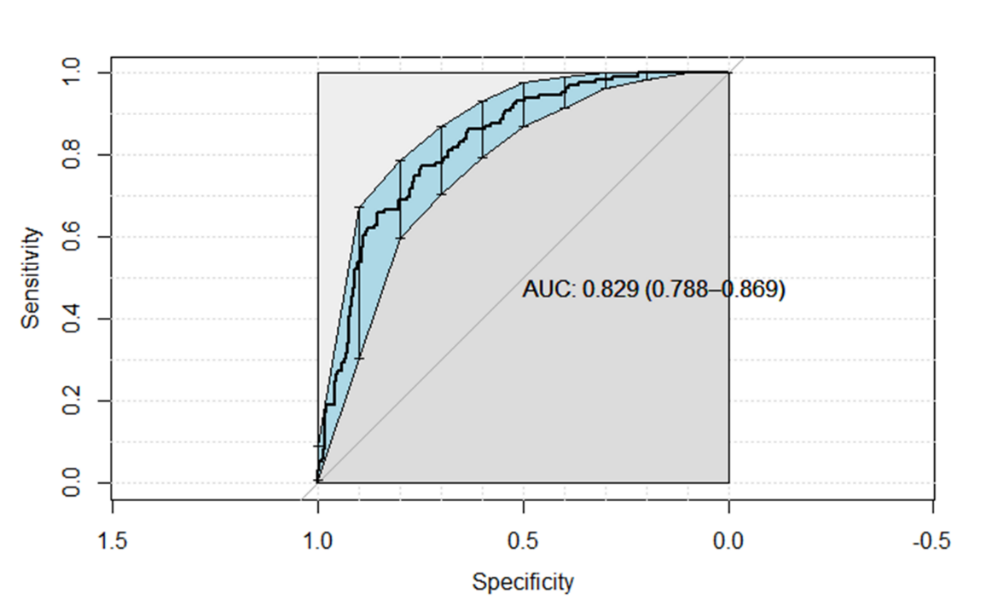


**e-Table 5**: Ranking of variables according to the information value to select important variables in a predictive model. Highly and somewhat predictive variables were included in the model.

## VARIABLES Information Value HSTRENGTH

## 1 Hospital type 4.041964e-03 Not Predictive

## 2 Male 9.730608e-03 Not Predictive

## 3 Influenza vaccine 2.719595e-02 Not Predictive

## 4 shock 1.025376e-01 Highly Predictive

## 5 Health worker 3.939070e-02 Somewhat Predictive

## 6 ACEI 5.176515e-02 Somewhat Predictive

## 7 ARB 1.407840e-02 Not Predictive

## 8 asthma 4.023492e-04 Not Predictive

## 9 COPD 4.605924e-02 Somewhat Predictive

## 10 Chronic Cardiac Dis 3.472710e-03 Not Predictive

## 11 Chronic Renal Dis 2.721800e-02 Not Predictive

## 12 Hematological Dis 2.144353e-02 Not Predictive

## 13 Pregnancy 2.253727e-03 Not Predictive

## 14 Obesity 2.452981e-03 Not Predictive

## 15 Diabetes 4.266095e-02 Somewhat Predictive

## 16 HIV 9.368383e-04 Not Predictive

## 17 Neuromuscular Dis 8.444705e-03 Not Predictive

## 18 Autoimmune Dis 5.088465e-04 Not Predictive

## 19 Coronary Dis 5.313002e-02 Somewhat Predictive

## 20 Hypertension 9.908557e-02 Somewhat Predictive

## 21 Infiltrates chest x-ray 3.931693e-02 Somewhat Predictive

## 22 Corticosteroids 4.460850e-03 Not Predictive

## 23 Antibiotics 7.133596e-05 Not Predictive

## 24 Empiric treatment 1.926115e-04 Not Predictive

## 25 Lopinavir/ritonavir 2.828696e-03 Not Predictive

## 26 interferon beta-1 1.351955e-02 Not Predictive

## 27 Hydroxychloroquine 8.557354e-03 Not Predictive

## 28 Tocilizumab 9.848787e-03 Not Predictive

## 29 O2 7.471572e-03 Not Predictive

## 30 Bacterial coinfection 6.317831e-03 Not predictive

## 31 HFNC 6.584694e-02 Somewhat Predictive

## 32 NIV 1.044679e-03 Not Predictive

## 33 MV 1.195287e-01 Highly Predictive

## 34 Myocardial Dysf. 1.020782e-01 Highly Predictive

## 35 Acute Kidney injury 4.267500e-01 Highly Predictive

## 36 Age 5.583901e-01 Highly Predictive

## 37 APACHE II 5.038235e-01 Highly Predictive

## 38 SOFA 3.964658e-01 Highly Predictive

## 39 Lactate dehydrogenase 1.623883e-01 Highly Predictive

## 40 WBC 5.118266e-02 Somewhat Predictive

## 41 Creatinine 2.605650e-01 Highly Predictive

## 42 CRP 5.374965e-02 Somewhat Predictive

## 43 PCT 1.590328e-01 Highly Predictive

## 44 Lactate 1.674733e-01 Highly Predictive

## 45 D Dimer 1.728036e-01 Highly Predictive

## 46 Ferritin 3.332789e-01 Highly Predictive

## 47 Gap antiviral 0.000000e+00 Not Predictive

## 48 PaO2/FiO2 6.116536e-02 Somewhat Predictive

## 49 Gap diagnostic 1.738805e-02 Not Predictive

## 50 Gap ICU 5.325043e-02 Somewhat Predictive

ACEI: Angiotensin Converting Enzyme Inhibitors; ARB: Angiotensin receptor blockers; WBC: White blood cells; HFNC: High Flow nasal cannula, NIV: non-invasive ventilation, MV: invasive mechanical ventilation, CRP: C-Reactive Protein; PCT: Procalcitonin ; GAP antiviral: Time from the symptoms onset to the first dose of antiviral, GAP diagnostic: Time from the symptoms onset to diagnosis, GAP ICU: time from the symptoms onset to ICU admission.

**e-Figure 5:** Categorized variables independently associated with ICU mortality


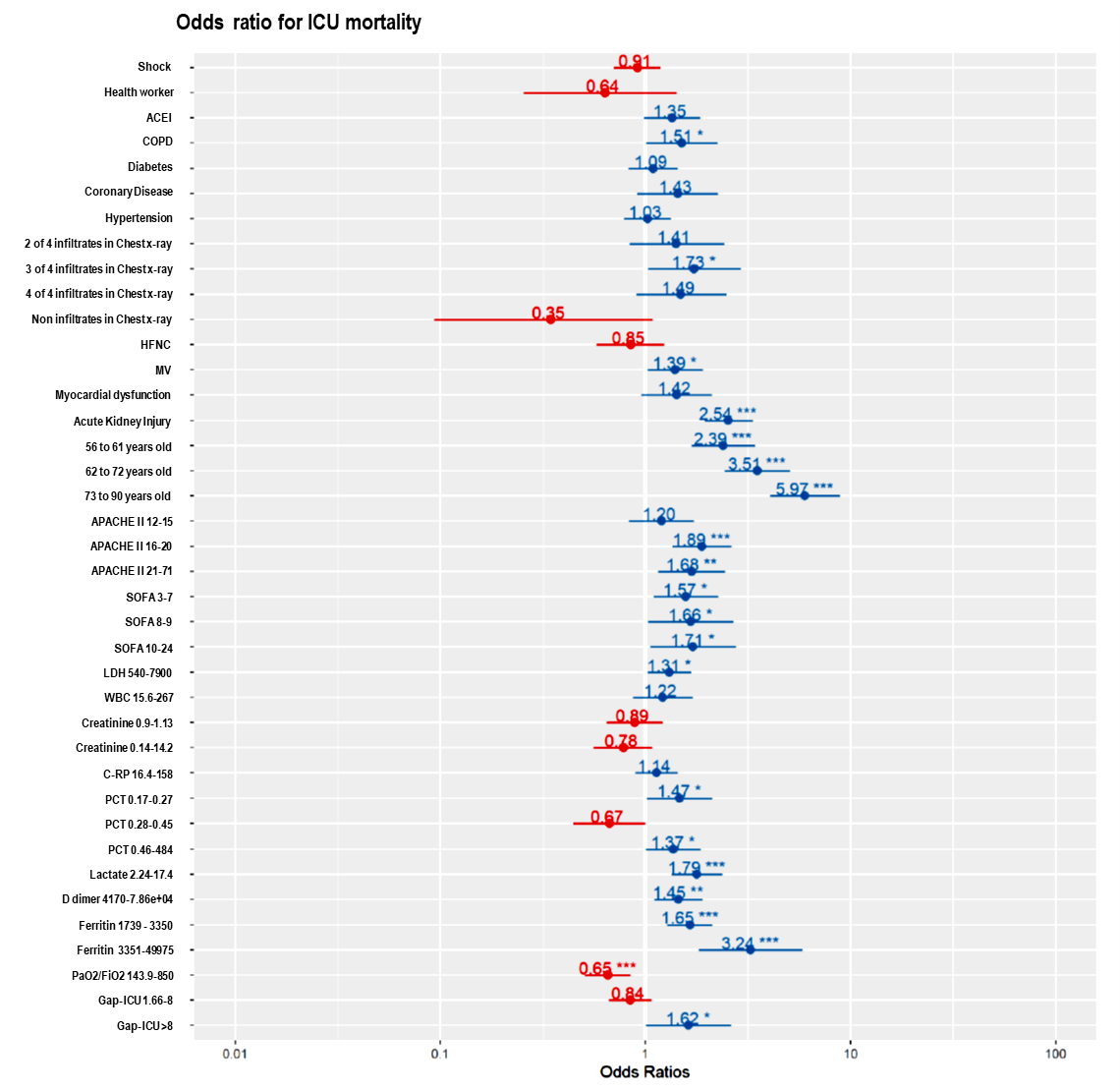


ACEI: Angiotensin Converting Enzyme Inhibitors; COPD; Chronic obstructive pulmonary disease; WBC: White blood cells; HFNC: High Flow nasal cannula, MV: invasive mechanical ventilation; APACHE II: Acute Physiology and Chronic Health Evaluation II; SOFA: Sequential Organ Failure Assessment; LDH: D-Lactate dehydrogenase; CRP: C-Reactive Protein; PCT: Procalcitonin; GAP ICU: time from the symptoms onset to ICU admission. *p<0.05, **p<0.01, ***p<0.001

**e-Figure 6**: Silhouette plot. The silhouette coefficient contrasts the average distance to elements in the same cluster with the average distance to elements in other clusters. Objects with a high silhouette value are considered well clustered, objects with a low value may be outliers. This index works well with k-medoids clustering, and is used to determine the optimal number of clusters. In our case, 3 clusters had the adequate silhouette width.
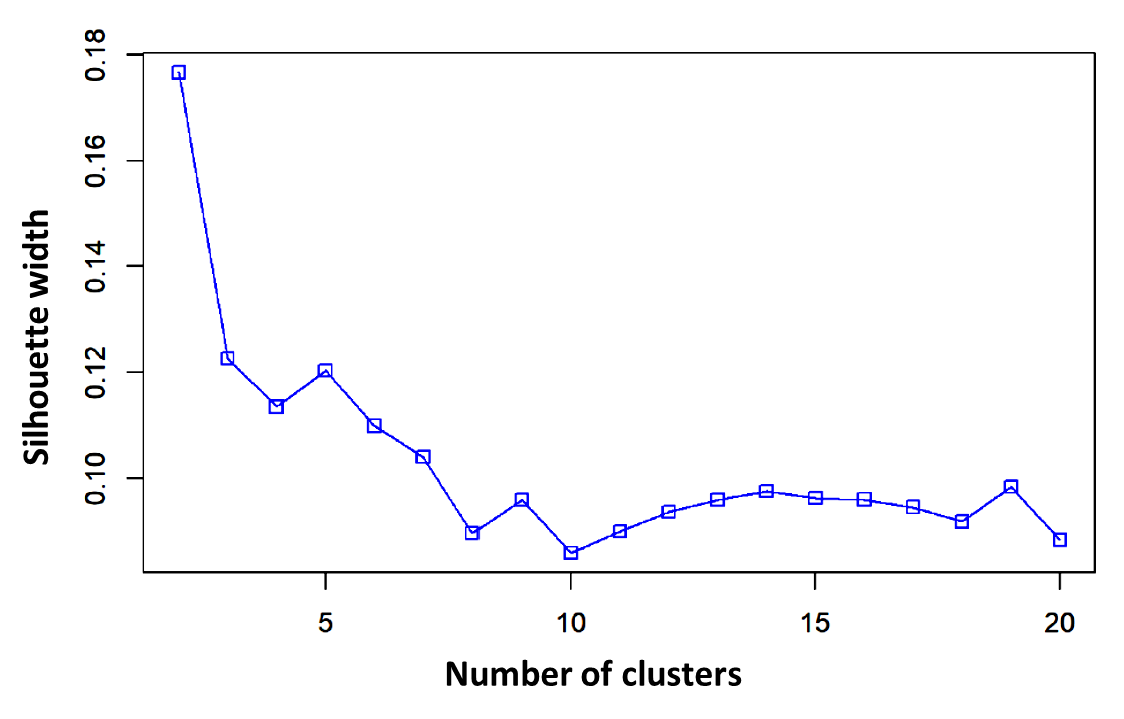


**e-Figure 7**: PAM Plot.

PAM is a per-partition clustering method that searches for "medoids" or prototypes, that is, cluster representative objects that have a minimum distance to the rest of the members of their cluster. In the PAM method the aim is to minimisation of the sum of dissimilarities between cluster members and their medoid. This difference in function to optimize makes PAM store to be more robust than k-means data sets with outliers. When working with the dissimilarity matrix there is more flexibility as to the type of data and distance measures that can be applied.


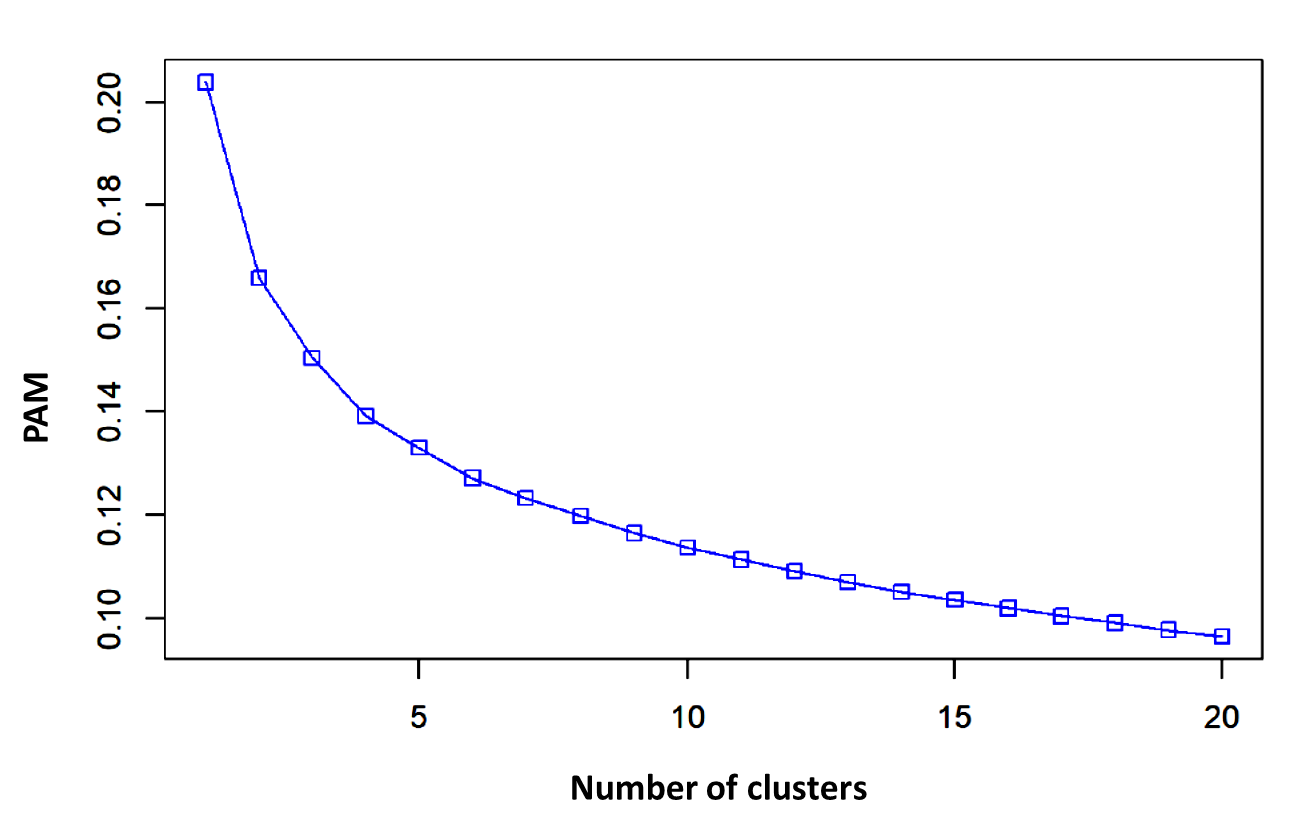


**e-Figure 8**: Plot of clinical phenotypes (clusters analysis) in a lower dimensional space using Principal Component Analysis.


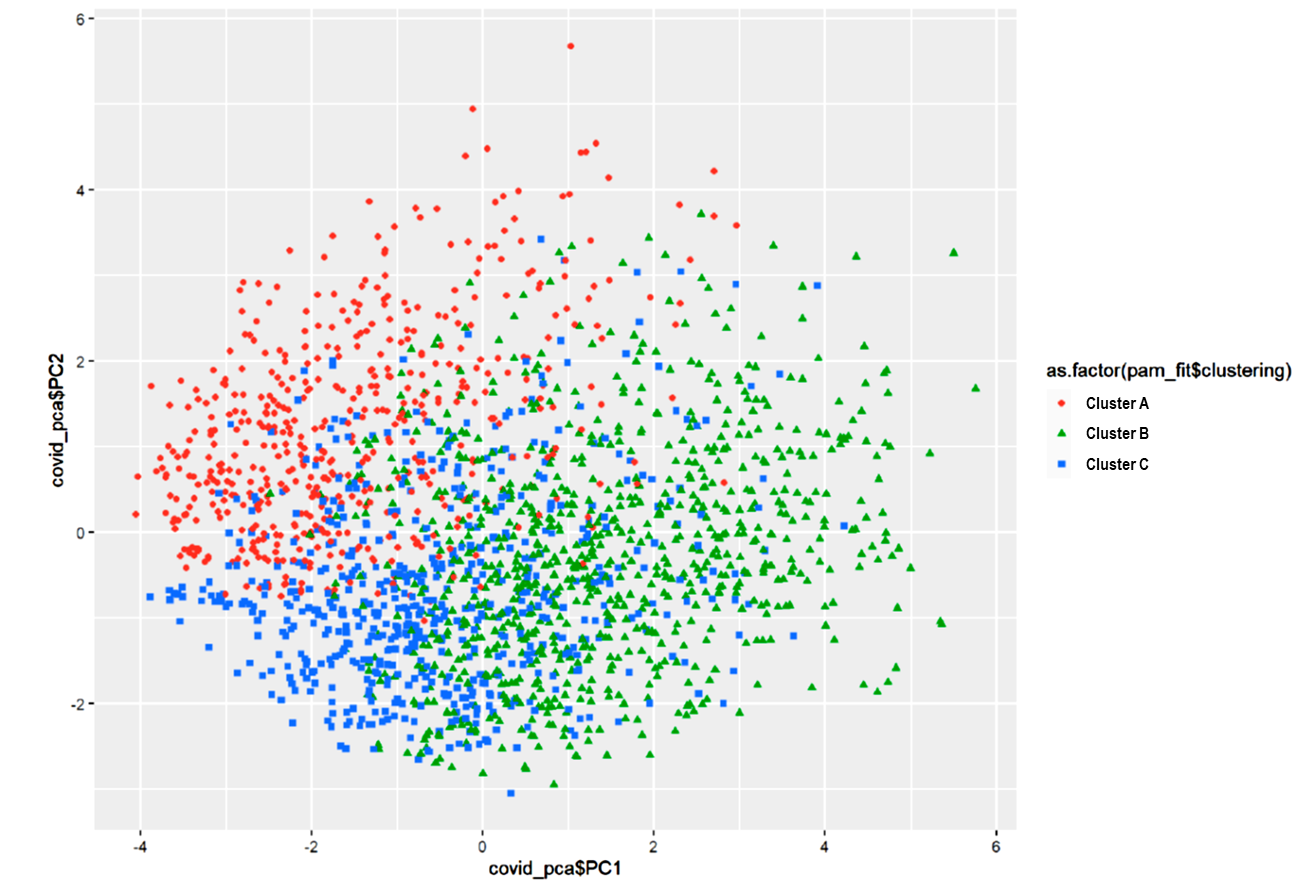


**e-Table 6**: Ranking of variables according to the information value to select important variables in each phenotype. Variables highlighted in red were included in each model.

|  | **A Phenotype** | **B Phenotype** | **C Phenotype** |
| --- | --- | --- | --- |
| **Variables** | IV PREDICTIVE | IV PREDICTIVE | IV PREDICTIVE |
| **## 1 Hospital type** | 9.132863e-03 No | 1.559446e-03 No | 2.944340e-02 No |
| **## 2 Male** | 1.444146e-04 No | 2.590207e-02 No | 3.869809e-03 No |
| **## 3 Influenza vaccine** | 9.431129e-02 Somewhat | 2.126550e-02 No | 3.765656e-02 Somewhat |
| **## 4 shock** | 3.964703e-02 Somewhat | 5.164118e-03 No | 4.618684e-04 No |
| **## 5 Health worker** | 7.215440e-02 Somewhat | 6.021809e-02 Somewhat | 9.813392e-03 No |
| **## 6 ACEI** | 1.684165e-01 Highly | 6.080522e-03 No | 1.076237e-02 No |
| **## 7 ARB** | 1.828984e-02 No | 2.404218e-04 No | 1.177667e-03 No |
| **## 8 asthma** | 1.227356e-02 No | 2.533545e-04 No | 1.092745e-04 No |
| **## 9 COPD** | 1.749341e-01 Highly | 7.302773e-02 Somewhat | 6.691157e-03 No |
| **## 10 Chronic Cardiac Disease** | 6.204765e-02 Somewhat | 1.492305e-03 No | 2.960268e-05 No |
| **## 11 Chronic Renal Disease** | 1.738024e-01 Highly | 1.492305e-03 No | 1.146307e-02 No |
| **## 12 Hematological Disease** | 2.654456e-02 No | 3.754947e-03 No | 4.883194e-02 Somewhat |
| **## 13 Pregnancy** | 9.222784e-03 No | 1.283035e-02 No | 0.000000e+00 No |
| **## 14 Obesity** | 3.637363e-02 Somewhat | 6.145638e-07 No | 2.066138e-04 No |
| **## 15 Diabetes** | 1.866242e-01 Highly | 5.704696e-03 No | 2.479443e-02 No |
| **## 16 HIV** | 1.819062e-02 No | 4.376645e-03 No | 8.041982e-05 No |
| **## 17 Neuromuscular Disease** | 3.325893e-02 Somewhat | 4.106090e-03 No | 4.524557e-03 No |
| **## 18 Autoimmune Disease** | 1.319137e-05 No | 7.433381e-03 No | 3.870564e-03 No |
| **## 19 Coronary Disease** | 1.340642e-01 Highly | 2.627084e-03 No | 1.221929e-01 Highly |
| **## 20 Hypertension** | 2.210587e-01 Highly | 1.186944e-02 No | 1.223492e-02 No |
| **## 21 Infiltrates chest x-ray** | 2.817873e-02 No | 1.564487e-01 Highly | 3.578773e-02 Somewhat |
| **## 22 Corticosteroids** | 1.229703e-03 No | 1.560437e-04 No | 1.635793e-03 No |
| **## 23 Antibiotics** | 6.889626e-03 No | 3.134299e-03 No | 2.975869e-04 No |
| **## 24 Empiric treatment** | 5.959459e-03 No | 4.937022e-03 No | 2.287937e-03 No |
| **## 25 Lopinavir/ritonavir** | 1.598323e-03 No | 5.711297e-02 Somewhat | 1.005827e-04 No |
| **## 26 interferon beta-1** | 2.656602e-02 No | 2.371890e-02 No | 8.530341e-03 No |
| **## 27 Hydroxychloroquine** | 4.276412e-02 Somewhat | 2.599639e-04 No | 3.468705e-02 Somewhat |
| **## 28 Tocilizumab** | 1.916296e-02 No | 2.610744e-03 No | 1.415470e-02 No |
| **## 29 O2** | 2.146274e-05 No | 3.402079e-02 Somewhat | 4.340230e-03 No |
| **## 30 HFNC** | 2.353027e-03 No | 7.516552e-04 No | 1.157880e-03 No |
| **## 31 NIV** | 1.070219e-02 No | 3.063022e-02 Somewhat | 3.737383e-05 No |
| **## 32 MV** | 3.057185e-03 No | 1.653186e-01 Highly | 2.370305e-08 No |
| **## 33 Myocardial Dysfunction** | 2.228904e-01 Highly | 6.177601e-02 Somewhat | 6.055540e-02 Somewhat |
| **## 34 Acute Kidney injury** | 6.306028e-01 Highly | 3.646921e-01 Highly | 2.337694e-01 Highly |
| **## 35 Age** | 6.513748e-01 Highly | 7.408417e-01 Highly | 4.622984e-01 Highly |
| **## 36 APACHE II** | 4.310389e-01 Highly | 2.986345e-01 Highly | 2.939476e-01 Highly |
| **## 37 SOFA** | 4.038444e-01 Highly | 2.953304e-01 Highly | 1.487510e-01 Highly |
| **## 38 Lactate dehydrogenase** | 5.366081e-02 Somewhat | 1.041057e-01 Highly | 3.504073e-02 Somewhat |
| **## 39 WBC** | 4.581520e-02 Somewhat | 2.181871e-03 No | 3.820653e-02 Somewhat |
| **## 40 Creatinine** | 5.615214e-01 Highly | 2.412052e-01 Highly | 9.217995e-02 Somewhat |
| **## 41 CRP** | 7.164503e-02 Somewhat | 8.547374e-02 Somewhat | 3.204018e-03 No |
| **## 42 PCT** | 3.048025e-01 Highly | 8.008344e-02 Somewhat | 0.000000e+00 No |
| **## 43 Lactate** | 6.190517e-02 Somewhat | 1.543924e-01 Highly | 2.290830e-01 Highly |
| **## 44 D Dimer** | 2.521748e-01 Highly | 4.644420e-01 Highly | 8.243056e-02 Somewhat |
| **## 45 Ferritin** | 4.180265e-01 Highly | 3.490296e-01 Highly | 1.818192e-01 Highly |
| **## 46 Gap antiviral** | 2.766619e-01 Highly | 0.000000e+00 No | 0.000000e+00 No |
| **## 47 PaO2/FiO2** | 0.000000e+00 No | 0.000000e+00 No | 1.018619e-01 Highly |
| **## 48 Gap diagnostic** | 0.000000e+00 No | 0.000000e+00 No | 0.000000e+00 No |
| **## 49 Gap ICU** | 6.204765e-02 Somewhat | 0.000000e+00 No | 7.482980e-02 Somewhat |

ACEI: Angiotensin Converting Enzyme Inhibitors; ARB: Angiotensin receptor blockers; WBC: White blood cells; HFNC: High Flow nasal cannula, NIV: non-invasive ventilation, MV: invasive mechanical ventilation, CRP: C-Reactive Protein; PCT: Procalcitonin; GAP antiviral: Time from the symptoms onset to the first dose of antiviral, GAP diagnostic: Time from the symptoms onset to diagnosis, GAP ICU: time from the symptoms onset to ICU admission.

**e-Figure 9**: Variables independently associated with ICU mortality rate in global model (all population) and in each phenotype observed in COVID-19 patients


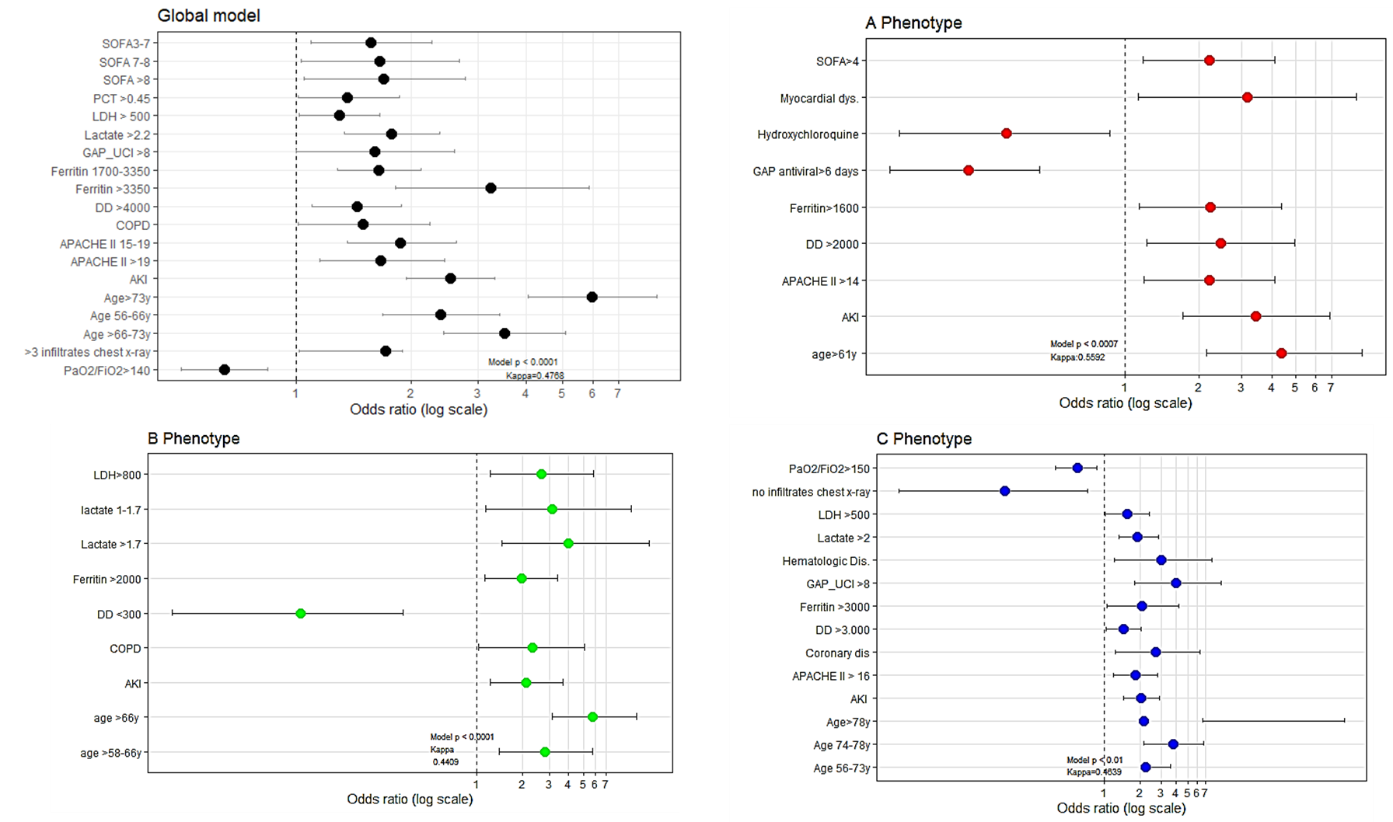

Supplement: Supplementary file 1 — Additional file 1. Supplementary online content. [file 13054_2021_3487_MOESM1_ESM.docx]
